# A Performance Evaluation of Large Language Models in Keratoconus: A Comparative Study of ChatGPT-3.5, ChatGPT-4.0, Gemini, Copilot, Chatsonic, and Perplexity

**DOI:** 10.3390/jcm13216512

**Published:** 2024-10-30

**Authors:** Ali Hakim Reyhan, Çağrı Mutaf, İrfan Uzun, Funda Yüksekyayla

**Affiliations:** Department of Ophthalmology, Faculty of Medicine, Harran University, 63100 Şanlıurfa, Türkiye; drmutaf1985@gmail.com (Ç.M.); irfanuzun@gmail.com (İ.U.); f.dilmen@hotmail.com (F.Y.)

**Keywords:** keratoconus, chatbots, large language models

## Abstract

**Background:** This study evaluates the ability of six popular chatbots; ChatGPT-3.5, ChatGPT-4.0, Gemini, Copilot, Chatsonic, and Perplexity, to provide reliable answers to questions concerning keratoconus. **Methods:** Chatbots responses were assessed using mDISCERN (range: 15–75) and Global Quality Score (GQS) (range: 1–5) metrics. Readability was evaluated using nine validated readability assessments. We also addressed the quality and accountability of websites from which the questions originated. **Results:** We analyzed 20 websites, 65% “Private practice or independent user” and 35% “Official patient education materials”. The mean JAMA benchmark score was 1.40 ± 0.91 (0–4 points), indicating low accountability. Reliability, measured using mDISCERN, ranged from 42.91 ± 3.15 (ChatGPT-3.5) to 46.95 ± 3.53 (Copilot). The most frequent question was “What is keratoconus?” with 70% of websites providing relevant information. This received the highest mDISCERN score (49.30 ± 4.91) and a relatively high GQS score (3.40 ± 0.56) with an Automated Readability Level Calculator score of 13.17 ± 2.13. Moderate positive correlations were determined between the website numbers and both mDISCERN (*r* = 0.265, *p* = 0.25) and GQS (*r* = 0.453, *p* = 0.05) scores. The quality of information, assessed using the GQS, ranged from 3.02 ± 0.55 (ChatGPT-3.5) to 3.31 ± 0.64 (Gemini) (*p* = 0.34). The differences between the texts were statistically significant. Gemini emerged as the easiest to read, while ChatGPT-3.5 and Perplexity were the most difficult. Based on mDISCERN scores, Gemini and Copilot exhibited the highest percentage of responses in the “good” range (51–62 points). For the GQS, the Gemini model exhibited the highest percentage of responses in the “good” quality range with 40% of its responses scoring 4–5. **Conclusions:** While all chatbots performed well, Gemini and Copilot showed better reliability and quality. However, their readability often exceeded recommended levels. Continuous improvements are essential to match information with patients’ health literacy for effective use in ophthalmology.

## 1. Introduction

Artificial Intelligence (AI) technologies are evolving rapidly and are expected to revolutionize the field of medicine [1]. When implemented appropriately, AI has the potential to enhance the efficiency and safety of medical practice by reducing clinician burnout and improving patient communication [2]. However, the medical field demands a high level of accuracy and reliability, since misinformation can lead to adverse health outcomes [3]. The advent of large language models (LLMs) has revolutionized the field of natural language processing (NLP), enabling machines to generate human-like and contextually appropriate responses. Models such as ChatGPT-3.5, ChatGPT-4.0, Gemini, Copilot, Chatsonic, and Perplexity have attracted significant attention for their potential applications across various domains, particularly in healthcare. However, the accuracy and reliability of LLMs in specific medical contexts remain underexplored.

Recent advances in AI have brought large language models, such as ChatGPT, to the forefront of ophthalmology. These models have demonstrated significant potential in assisting with symptom analysis, treatment recommendations, and patient education as well as exhibiting notable performance in disease diagnosis and symptom triaging [4]. However, their clinical implementation faces challenges, including data security concerns, ethical considerations, and inherent limitations such as outdated training data and the potential for generating misinformation [5]. Despite these hurdles, the integration of large language models in ophthalmology holds significant promise for enhancing diagnostic accuracy and improving patient care [6]. A balanced approach, combining the capabilities of AI with human clinical judgment, is crucial in order to fully leverage these benefits while mitigating the risks.

Keratoconus is a progressive eye disease characterized by the thinning and bulging of the cornea, leading to visual impairment. Patients and caregivers frequently seek information regarding the symptoms, diagnosis, and therapeutic options for this condition that affects a significant part of the population [7]. Due to the complexity and specificity of medical information, it is crucially important to evaluate the performance of LLMs in providing accurate and reliable answers to questions related to keratoconus. Obtaining early and accurate information is essential for effective management and treatment. This trend underscores the importance of evaluating the quality of information provided by LLMs, which are increasingly being used to answer health-related queries.

The objective of this study was threefold. First, we set out to systematically evaluate the accountability of websites that serve as sources for keratoconus-related questions. Secondly, we intended to assess the capability of six popular chatbots (ChatGPT-3.5, ChatGPT-4.0, Gemini, Copilot, Chatsonic, and Perplexity) in providing reliable and accurate responses to these questions, comparing their performance with each other. Finally, we sought to analyze the readability of the responses generated by these LLMs using nine validated readability scales, assessing their potential utility in delivering accessible patient education in ophthalmology.

## 2. Materials and Methods

### 2.1. Ethics

Since the LLMs used in this study are public applications and no patients were involved, ethics committee approval was not required.

### 2.2. Data Collection and Search Strategy

All Google searches used in data collection were executed using a clean-installed Google Chrome (Menlo Park, CA, USA) browser in Incognito Mode. In order to avoid bias from previous searches and targeted search results based on geography, we disabled all location filters, advertisements, and sponsored results. The search terms used were “Keratoconus FAQ”, and the “People also ask” box was used to obtain FAQs generated by Google’s machine learning algorithms.

### 2.3. Question Selection and Categorization

The first 20 websites were reviewed (Supplemental Table S1). The 20 most frequently asked questions concerning keratoconus were selected by two experienced ophthalmologists (A.H.R., Ç.M.), who subsequently transformed similar question patterns into a common question template. Websites used to answer each of the 20 FAQs in this study were first categorized according to the information source: (1) private practice or independent user or (2) official patient education materials published by a national organization (such as the American Academy of Ophthalmology).

### 2.4. JAMA Accountability Analysis

All websites were evaluated for accountability (scores of 0–4) using JAMA benchmarks. According to JAMA guidelines, a website containing patient education materials should (1) include all authors and their relevant credentials, (2) list references, (3) provide disclosures, and (4) provide the date of the most recent update. The scoring was conducted by three experienced ophthalmologists (A.H.R., Ç.M., and İ.U.).

### 2.5. Large Language Models (LLMs)

The LLMs were trained on extensive bodies of text data, including books, scholarly articles, and web pages, covering a wide array of subjects including medicine, sports, and politics. The LLM models employed were ChatGPT-3.5, ChatGPT-4, Gemini, Copilot, Chatsonic, and Perplexity. These were asked 20 FAQs related to ‘keratoconus’, and their responses were recorded (Supplemental Table S2).

### 2.6. Evaluation of LLM-Chatbot Responses

As shown in Table 1, mDISCERN is a scoring system developed by Oxford University, consisting of three parts and 16 questions and used to evaluate the reliability and quality of online health information. The mDISCERN scoring system result range is 15–75, and the results are classified as excellent (63–75 points), good (51–62), reasonable (39–50), poor (27–38), or very poor (15–26). The Global Quality Scale (GQS) was applied to assess the quality of LLM responses. Accordingly, 1 point indicates poor quality, and 5 points indicate excellent quality (Table 1). Additionally, this scale was also used for quality classification, 1–2 points representing low quality, 3 points moderate quality, and 4–5 points high quality.

The LLM-Chatbots responses were evaluated and scored in a double-blinded manner by three experienced ophthalmologists. The LLM-Chatbot responses represented the average scores given by three experienced ophthalmologists using DISCERN (15–75 points) and GQS (1–5 points) (A.H.R., Ç.M., and İ.U.). A consensus score was then determined.

### 2.7. Readability Analysis

Each of the 20 websites that provided answers to the 20 FAQs examined in this study was evaluated for readability using readability assessments: Flesch Reading Ease (FRE), Flesch–Kincaid grade level (FKGL) and the Automated Readability Level Calculator (ARLC) [8,9,10].

### 2.8. Statistical Analysis

Statistical analyses were conducted using R software (Version 4.1.1, R Foundation, Vienna, Austria). Descriptive statistics were used to categorize the sources of online information regarding keratoconus. Categorical variables were expressed as numbers and percentages. Differences in the length and readability of responses across the LLM-Chatbots were compared using one-way ANOVA and Tukey’s honestly significant post hoc test since the samples met parametric assumptions. To complement the *p*-values and provide a measure of the effect size, we calculated Eta Squared (η^2^). Relationships between the data were evaluated with a two-tailed Pearson’s χ^2^ test. A *p*-value less than 0.05 was considered statistically significant.

## 3. Results

### 3.1. Frequently Asked Questions After Google Searches for ‘Keratoconus’

Table 2 shows the distribution of website categories and their JAMA benchmark scores in terms of LLM accuracy in providing keratoconus-related information. Seventy-five percent (15) of the 20 websites were “Private practice or independent user” and 25% (5) were “Official patient education materials”.

### 3.2. JAMA Accountability Scores for Webpages to Keratoconus-Related FAQs

The mean JAMA benchmark score for all websites was 1.40 ± 0.91 out of a maximum of 4. The mean score for “Private practice or independent user” websites was 1.0 ± 0.63, while that for “Official patient education materials” was 2.6 ± 0.48. In terms of JAMA scores, 13 websites met authorship criteria, 4 met attribution criteria, 5 met disclosure criteria, and 4 met currency criteria. Most websites (45%) scored only 1, indicating poor adherence to JAMA guidelines. None of the websites scored 4. Three websites (15%) achieved scores of 3, representing moderate accountability. Five websites (25%) scored 2, and three websites (15%) scored 0.

### 3.3. Average Score for Each Question

Table 3 evaluates the performance of LLMs in answering keratoconus-FAQ using mDISCERN, GQS, and ARLC scores. The most frequently addressed question was “What is keratoconus?” (on 70% of websites), which received the highest mDISCERN score (49.30 ± 4.91), a high GQS score (3.40 ± 0.56), and an ARLC score of 13.17 ± 2.13. Other questions exhibited lower coverage. For example, “How do patients with keratoconus see?” (on 15% of websites) received an mDISCERN score of 44.9 ± 3.40, a GQS score of 3.12 ± 0.60, and an ARLC score of 14.17 ± 2.85. Scores for “Can keratoconus go away on its own?” (on 15% of websites) were mDISCERN 44.3 ± 3.56, GQS 2.85 ± 0.42, and ARLC 14.5 ± 1.64. “What should be considered after keratoconus surgery?” (on 15% of websites) received an mDISCERN score of 46.32 ± 2.84, a GQS score of 3.15 ± 0.40 and an ARLC score of 13.67 ± 2.06.

Moderate positive correlations were observed between the number of websites and both mDISCERN (*r* = 0.265, *p* = 0.25) and GQS scores (*r* = 0.453, *p* = 0.05), indicating higher quality and reliability for FAQs. However, a weak negative correlation was found between the number of websites and ARLC scores (*r* = −0.151, *p* = 0.55), suggesting that readability is not strongly correlated with the number of websites addressing a particular question.

The score for each question was calculated by averaging the scores of the large language models’ (ChatGPT-3.5, ChatGPT-4.0, Gemini, Copilot, Chatsonic, and Perplexity) ARLC; processing the text through eight popular readability formulas (Linsear Write Formula, SMOG Index, Coleman–Liau Index, Flesch–Kincaid Grade Level, Gunning Fog Index, Flesch Reading Ease Formula, Automated Readability Index, FORCAST Readability Formula) and averaging out the results to yield an approximate reading difficulty score.

### 3.4. Reliability

#### 3.4.1. mDISCERN Score

All LLMs performed reasonably well with Gemini, Copilot, and Perplexity exhibiting higher reliability. The lowest mDISCERN score was 42.91 ± 3.15 (ChatGPT-3.5), and the highest was 46.95 ± 3.53 (Copilot). The differences were statistically significant (*p* < 0.05).

#### 3.4.2. GQS Score

The lowest GQS score was 3.02 ± 0.52, which was observed in the ChatGPT-3.5 model, and the highest was 3.31 ± 0.64, which was observed in the Gemini model. The differences between these models were not significant with a *p*-value of 0.34. Gemini and Copilot again achieved higher scores, indicating better overall quality.

#### 3.4.3. Readability Indexes

In terms of the readability indexes of all texts, Table 4 shows how the various models performed in the context of text comprehensibility. The texts were clearly generally difficult to read and were suitable for readers educated to high school or college level. The *p*-values for all indexes were <0.05, and it may therefore be concluded that the differences between the texts were statistically significant. Gemini emerged as the easiest readable text, having received the lowest score on most readability indexes. More specifically, the low scores on the FRE and FKGL indexes suggest that the texts were simpler and more comprehensible. ChatGPT-3.5 and Perplexity emerged as the most difficult readable texts, exhibiting the highest scores on most readability indexes.

#### 3.4.4. Response Length

Copilot generated the longest responses (16 ± 4.69 sentences) and Perplexity the shortest (6.9 ± 4.29). ChatGPT-4 produced the most words (285.9 ± 60.76) and Perplexity the fewest (127.8 ± 51.96). Chatsonic exhibited the highest character count (1985.2 ± 491.80) and syllable count (564.05 ± 133.18), the lowest values for both being determined in Perplexity (893.6 ± 379.47 characters and 251.6 ± 106.22 syllables). ChatGPT-4 exhibited the highest words per sentence ratio (20.43 ± 3.39) and Copilot the lowest (11.3 ± 2.52). Chatsonic registered the highest syllables per word ratio (2.01 ± 0.10) and Gemini the lowest (1.86 ± 0.09). All differences were statistically significant (*p* < 0.05).

#### 3.4.5. Score Distributions of mDISCERN Scale and Quality Classification

Table 5 presents the mDISCERN score distribution and quality classification of keratoconus responses from the various different LLMs. Most models (75–95%) scored in the “Reasonable” range (39–50 points). Gemini and Copilot achieved the highest “Good” range scores (51–62 points) at 30% and 20%, respectively. However, no model achieved the “Excellent” range (63–75 points). Perplexity and Chatsonic exhibited the highest “Poor” range scores (27–38 points) at 5% and 10%, respectively.

GQS exhibited moderate quality with most models scoring in the range of 3–3.5. Gemini achieved the highest “good” quality responses (40% scoring 4–5 points). Copilot and Chatsonic also registered significant “good” quality responses (30%). ChatGPT-3.5 and ChatGPT-4.0 exhibited the lowest “good” quality responses at 15% and 10%, respectively.

## 4. Discussion

This study evaluated the efficacy of six LLMs in terms of accurately responding to medical queries by comparing their performance on common keratoconus-related questions sourced from Google searches. The findings indicate that LLM-Chatbots have the potential to provide comprehensive responses to keratoconus-related inquiries.

LLMs can provide keratoconus patients with up-to-date, evidence-based information, facilitating rapid access to the latest therapeutic options and research findings. Patients can use LLMs for a better understanding of their condition and to make informed healthcare decisions. Accurate and comprehensible information from LLMs can enhance patient adherence to treatment plans and alleviate concerns, improving their emotional well-being. LLMs also have the potential to empower keratoconus patients by equipping them with the knowledge required for active participation in their healthcare journeys.

This study investigated practical scenarios in which concerned patients might seek assistance from emerging resources. To the best of our awareness, this is the first study to evaluate LLM responses to keratoconus-related queries. The research builds on previous studies examining the applicability of LLM chatbots, such as ChatGPT, across various medical subspecialties. Prior research has explored the use of LLMs for providing medical information, patient education, and diagnostic and treatment recommendations albeit with mixed results [11,12]. One significant cause for concern is the potential for misinformation in medical chatbots, which can be manipulated by special interest groups [13]. AI models are designed to generate content based on probable word sequences rather than producing factual answers. While generative AI chatbots can debunk misinformation, they can also spread falsehoods if not regularly updated with the latest scientific evidence [14]. For example, Lim et al. reported that ChatGPT-3.5 incorrectly stated that “Atropine eye drops are a new treatment for myopia and their optimal dosage has not yet been determined” [15]. Giuffrè et al. evaluated LLMs in the context of digestive diseases and concluded that despite their potential, their current accuracy and reliability are inadequate for clinical use [16]. Conversely, LLMs such as ChatGPT and Google Bard have exhibited impressive medical knowledge and capabilities, proving beneficial for patient communication [17,18,19].

Recent studies evaluating the performance of various LLM-linked chatbots in healthcare applications have identified distinct strengths and weaknesses. Huo et al. assessed chatbots such as ChatGPT-4, Copilot, Google Bard, and Perplexity AI for their recommendations on the surgical management of gastroesophageal reflux disease. Google Bard provided the most accurate recommendations for both physicians and patients, closely followed by ChatGPT-4, while Copilot and Perplexity AI demonstrated lower accuracy rates [20]. Similarly, Lim et al. evaluated the performance of LLM-linked chatbots, including ChatGPT-3.5, Claude, Gemini, and CoPilot, for perioperative advice on abdominoplasty. Claude emerged as the most reliable, followed by ChatGPT-3.5, while CoPilot and Gemini exhibited lower reliability [21]. Masalkhi et al. compared the capabilities of LLMs, particularly Gemini AI and ChatGPT, in the context of healthcare applications. Gemini AI excelled in language understanding and multimodal processing, while ChatGPT demonstrated strong medical knowledge, visual analysis, and personalized guidance capabilities [22]. Duran et al. evaluated the performance of various LLMs, including ChatGPT-4, Gemini, and Copilot, based on the readability, clarity, and accuracy of their medical information responses for cosmetic surgery procedures. ChatGPT-4 emerged as superior in generating patient-facing materials with precise and comprehensive medical information, illustrating the distinct strengths of different LLMs in medical communication [23].

In the field of ophthalmology, LLM chatbots have exhibited promise in addressing common patient queries concerning eye health [24]. Cohen et al. determined that human responses to ophthalmology-based questions contained a similar rate of incorrect or inappropriate material (27%) as also reported by Bernstein et al. [25,26]. However, AI responses in the current study were more accurate (94%) than those provided by ChatGPT in Bernstein et al.’s study (77%) [26].

In this study, responses from private practice or independent organization websites (65%, *n* = 13) registered a mean JAMA accountability score of 1.5 ± 0.68, indicating infrequent adherence to JAMA criteria. Official patient education materials from national organizations (35%, *n* = 7) had a slightly higher mean score of 1.57 ± 0.75, suggesting marginally better compliance. The overall mean JAMA benchmark score was 1.5 ± 0.68, indicating low accountability across the websites. This trend is consistent with numerous previous studies. A comprehensive analysis of five studies regarding the readability and accountability of online ophthalmology patient education materials reported a mean JAMA accountability score of 1.13 with a standard deviation of 1.15, reflecting substantial deficiencies in both quality and accountability [27,28,29,30,31]. These findings highlight the need for improved standards in creating and disseminating online patient education materials.

When considering keratoconus-related FAQs, metrics such as mDISCERN, GQS, and ARLC scores provide insights into the performance of LLMs in the medical sphere. The question “What is keratoconus?”, addressed by 70% of websites, registered the highest mDISCERN and GQS scores. In contrast, less frequently addressed questions such as “Are there multiple forms of keratoconus?” (15%) received lower mDISCERN scores. Similarly, “Does keratoconus cause eye pain?” (20%) and “Can keratoconus go away on its own?” (15%) registered lower GQS scores. The ARLC score, indicating readability, exhibited less variability, with most questions scoring between 12 and 16. For instance, “What is keratoconus?” achieved an ARLC score of 13.17 ± 2.13, while “Can LASIK or RK surgery cause keratoconus?” (25%) scored 16.33 ± 2.65. These findings highlight the importance of question frequency in determining the response quality and the potential of LLMs to provide high-quality, reliable medical information, especially for frequently asked questions. However, readability does not exhibit strong correlation with the number of websites addressing a question.

mDISCERN indices evaluate the performance of LLMs in providing medical information, assessing the informativeness, accuracy, and safety of the content. Wilhelm et al. identified significant quality differences among LLMs with notable variability in mDISCERN scores. The Claude-instant-v1.0 model received the highest score, and Bloomz received the lowest [32]. The present study indicates that although all LLMs performed reasonably well, their ability to provide accurate and reliable medical information differs significantly. Models such Gemini and Copilot scored higher, suggesting better performance. The significant variability in mDISCERN scores underscores the need for continuous improvement and validation. Standardized evaluation metrics and rigorous testing protocols are essential for assessing AI model performance and identifying potential areas for improvement.

The present study evaluated the quality and reliability of LLM responses to questions related to keratoconus. However, it is crucially important to consider significant factors such as repeatability, robustness, and models confidence, since these are essential in evaluating the reliability of such models. These factors were examined in a comprehensive study by Krishna et al. in the field of radiology [33]. Those authors found that the GPT-3.5 and GPT-4 models demonstrated moderate internal consistency (κ = 0.64 and κ = 0.78, respectively), exhibited limited robustness against misleading inputs, and tended to overestimate their own capabilities. Notably, the higher rate of response alteration in GPT-4 (97.3%) compared to GPT-3.5 (71.3%) suggests that even more advanced models may be sensitive to external influences. Similarly, in Kochanek et al.’s study involving hearing tests, the accuracy rate of 65–69% and repeatability of 85–88% for ChatGPT-4 demonstrate the potential of LLMs in providing medical information while also indicating that the use of these technologies in clinical applications remains limited and requires careful consideration [34].

The mDISCERN score distribution in this study reveals that Gemini and Copilot performed better in the “good” range (51–62 points) compared to other LLMs, which was probably due to superior training data, fine tuning, or algorithms. Conversely, Perplexity and Chatsonic registered the highest percentage of responses in the “Poor” range (27–38 points), indicating potential weaknesses due to less comprehensive training and suboptimal fine tuning. These findings suggest that while LLMs can generate reasonably reliable medical information, there is a significant gap in achieving high reliability across all models. No model reached the “Excellent” range in mDISCERN scores, indicating that current LLMs are not yet capable of providing highly reliable information for all questions. Onder et al. evaluated ChatGPT-4 responses concerning hypothyroidism during pregnancy using DISCERN tools, reporting that most responses were either fair (78.9%) or good (21.1%) [35]. This highlights the model’s capability to generate dependable information in most instances. The performance differences among LLMs emphasize the need for ongoing research and development in order to enhance the reliability and quality of information generated by these models.

Evaluating LLMs using the GQS provided valuable insights into the quality of medical information generated by them. Although no significant differences were observed among LLMs, models such as Gemini and Copilot consistently scored higher, indicating better overall quality and more robust mechanisms for generating accurate content. Ostrowska et al. evaluated the reliability and safety GQS of LLMs in the context of laryngeal cancer, describing ChatGPT 3.5 as the most successful model [36]. This emphasizes the need for model-specific evaluations in order to identify the best-performing models for particular medical spheres.

GQS score analysis revealed varying levels of quality in medical information produced by LLMs. The majority of models scored in the 3–3.5 range on a five-point scale, indicating moderate quality. Gemini emerged as the top performer with 40% of its outputs in the “good” quality range (4–5 points). Copilot and Chatsonic also performed well with 30% of their responses in the “good” range. In contrast, ChatGPT models (3.5 and 4.0) achieved lower rates of “good” quality responses (15% and 10%, respectively). In contrast to our findings, Onder et al. reported that 84.2% of ChatGPT-4’s responses regarding hypothyroidism during pregnancy were of high quality, which was followed by 10.5% medium-quality responses [35]. This discrepancy suggests that the specific medical sphere or the nature of the questions in the present study may have been particularly challenging for these models, which is a subject warranting further investigation at a later date.

**Figure 1:**
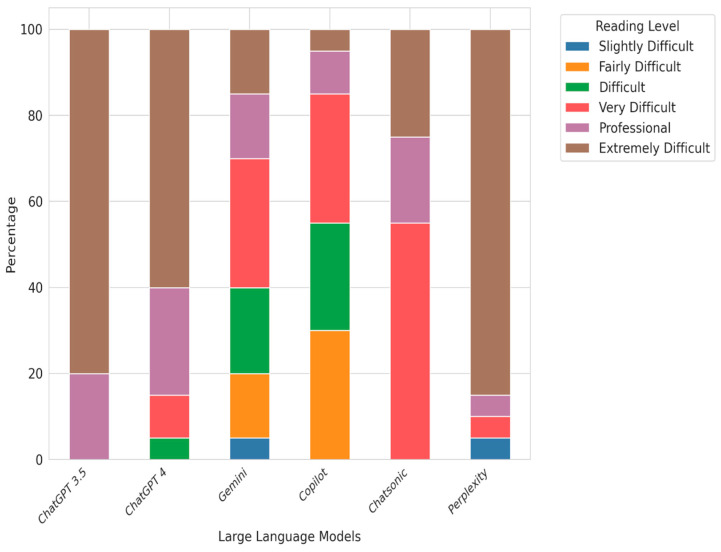
Comparative analysis of reading level difficulty (percentage values) across six large language models (ChatGPT-3.5, ChatGPT-4, Gemini, Copilot, Chatsonic, and Perplexity) using the Average Reading Level Consensus calculator (ARLC) for frequently asked questions on keratoconus.

**Figure 2:**
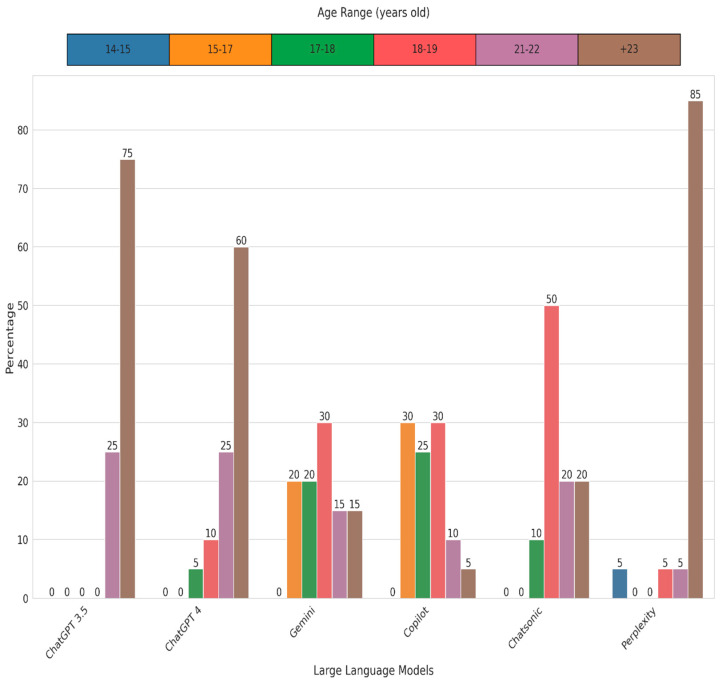
Comparative analysis of age range difficulty (percentage values) by six large language models (ChatGPT-3.5, ChatGPT-4, Gemini, Copilot, Chatsonic, and Perplexity) using the Average Reading Level Consensus calculator (ARLC) for frequently asked questions on keratoconus.

Although our expert evaluators preferred chatbot responses, their readability frequently exceeded the American Medical Association’s (AMA) recommendation of a sixth-grade reading level for patient education materials. Using eight popular readability formulae, the final ARLC scores indicated the following reading levels: ChatGPT-3.5, ChatGPT-4, and Perplexity were rated as extremely difficult, Gemini and Copilot as difficult, and Chatsonic as very difficult. The corresponding grade levels were ChatGPT-3.5, ChatGPT-4, Perplexity at the college graduate level, Gemini and Copilot at the twelfth grade level, and Chatsonic at college entry level (Figure 1 and Figure 2). These findings align with previous research showing that chatbot-generated patient education information is frequently written at reading levels significantly exceeding the comprehension of the average patient [25,37].

Research indicates that tailoring patient education materials to patients’ health literacy levels can significantly enhance compliance and optimize health outcomes [38]. A scoping review of visual aids in health literacy reported that materials intended for individuals with low literacy levels significantly improved health literacy outcomes, including medication adherence and comprehension [39].

While some chatbots, such as ChatGPT-4 and Chatsonic, produce detailed and complex responses, others, including Perplexity, generate shorter and simpler answers. These differences in response length and complexity highlight the varying capabilities of LLM-Chatbots in addressing keratoconus-related FAQs. This information is crucially important for selecting an appropriate chatbot for specific informational needs, particularly in medical and educational contexts in which the depth and clarity of information are paramount.

The adaptability of chatbots to user requests is significant for their potential application in ophthalmology. Despite challenging reading levels, providing patient education materials remains highly beneficial. This study demonstrates the usefulness of chatbots in providing keratoconus-related information for patients. Ophthalmologists report a loss of efficiency due to excessive time spent on non-clinical tasks. Chatbots can help alleviate this burden. A semi-supervised model, in which the ophthalmologist reviews AI-generated responses, represents the future of AI and can be highly beneficial tool for ophthalmologists.

This study has several limitations that need to be considered. First, we did not assess the speed of response or the consistency of LLM responses when the same question was posed multiple times, which might offer insights into their efficiency and reliability in real-world applications. Additionally, we did not focus on factors such as reproducibility, robustness, and the model’s confidence in its own responses while evaluating the quality and reliability of LLM responses to keratoconus-related questions. Furthermore, the questions were sourced from Google, and we did not investigate how patients interpreted these responses, which might impact on the practical applicability of the findings.

## 5. Conclusions

LLMs can provide comprehensive and accurate responses to keratoconus-related queries, enhancing patient adherence, decision making, and emotional well-being. However, the performance of the different LLM chatbots varies in terms of quality, reliability, and readability. While all LLMs performed commendably, Gemini and Copilot emerged as superior in providing reliable and high-quality information with Gemini demonstrating the best readability. In contrast, ChatGPT-3.5 and Perplexity produced the most difficult-to-read texts, potentially hindering patient comprehension. Tailoring information to patients’ health literacy levels is crucial. The continuous improvement and validation of LLM chatbots is essential together with standardized evaluation metrics and rigorous testing protocols. A semi-supervised model, in which an ophthalmologist reviews AI-generated responses, represents a promising approach to the integration of AI in ophthalmology, potentially reducing the burden on healthcare professionals.

## Figures and Tables

**Table 1:** The mDISCERN and GQS content and readability indexes.

The mDICERN Scoring System	Total Score (15–75 Points)
1. Are the aims clear?	1–5 points
2. Does it achieve its aims?	1–5 points
3. Is it relevant?	1–5 points
4. Is it clear what sources of information were used to compile the publication (other than the author or producer)?	1–5 points
5. Is it clear when the information used or reported in the publication was produced?	1–5 points
6. Is it balanced and unbiased?	1–5 points
7. Does it provide details of additional sources of support and information?	1–5 points
8. Does it refer to areas of uncertainty?	1–5 points
9. Does it describe how each treatment works?	1–5 points
10. Does it describe the benefits of each treatment?	1–5 points
11. Does it describe the risks of each treatment?	1–5 points
12. Does it describe what would happen if no treatment is used?	1–5 points
13. Does it describe how the treatment choices affect overall quality of life?	1–5 points
14. Is it clear that there may be more than 1 possible treatment choice?	1–5 points
15. Does it provide support for shared decision making?	1–5 points
16. Based on the answers to all of these questions, rate the overall quality of the publication	1–5 points
Global Quality Score	Score
Poor quality, very unlikely to be of any use to patients	0–1 Points
Poor quality but some information present, of very limited use to patients	0–1 Points
Suboptimal flow, some information covered but important topics missing, somewhat useful	0–1 Points
Good quality and flow, most important topics covered, useful to patients	0–1 Points
Excellent quality and flow, highly useful to patients	0–1 Points
Readability Indexes	
Flesch Reading Ease (FRE)	206.835 − (1.015 (W/S)) − (84.6 × (S/W)
Flesch–Kincaid grade level (FKGL)	0.39 × (W/S) + 11.8 × (B/W) − 15.59
Average Reading Level Consensus Calculator (ARLC)	Based on [8,9,10] above popular readability formulas, your text yielded a final result

B: number of syllables, W: number of words, S: number of sentences.

**Table 2:** Evaluation of web resources for frequently asked questions about keratoconus: categories determined by three experienced ophthalmologists and JAMA credibility scores used to assess information reliability (A.H.R., Ç.M., and İ.U.).

Website Category	Number *n* (%)	JAMA Benchmarks (Mean Score ± SD)
Private practice or independent user	15 (75%)	1.0 ± 0.63
Official patient education materials published by a national organization	5 (25%)	2.6 ± 0.48
Total	20 (100%)	1.4 ± 0.91
JAMA Benchmarks of Website	Score	Number *n* (%)
Authorship	13	
Attribution	4	
Disclosure	5	
Currency	4	
4.0		0 (0%)
3.0		3 (15%)
2.0		5 (25%)
1.0		9 (45%)
0.0		3 (15%)

**Table 3:** Comprehensive analysis of frequently asked questions about keratoconus based on web resources: frequency of questions, mDISCERN scores, Global Quality Scores (GQSs), and Average Reading Level Consensus calculator (ARLC) scores assigned by three experienced ophthalmologists (A.H.R., Ç.M., and İ.U.).

No	Question	Number of Websites *n* (%)	mDISCERN (Mean ± SD)	GQS (Mean ± SD)	ARLC (Mean ± SD)
1.	What Is Keratoconus?	14 (70%)	49.30 ± 4.91	3.4 ± 0.56	13.17 ± 2.13
2.	What Are the Symptoms of Keratoconus?	10 (50%)	43.6 ± 2.55	3.1 ± 0.00	12.33 ± 2.33
3.	How Do Patients with Keratoconus See?	3 (15%)	44.9 ± 3.40	3.12 ± 0.60	14.17 ± 2.85
4.	How Can Keratoconus Affect My Life?	6 (30%)	46.1 ± 3.31	3.15 ± 0.40	14.5 ± 1.37
5.	How Common Is Keratoconus?	7 (35%)	44.10 ± 3.18	3.15 ± 0.74	13.67 ± 1.96
6.	What Causes Keratoconus?	14 (70%)	44.1 ± 6.39	2.98 ± 0.61	15.5 ± 1.37
7.	Does Keratoconus Cause Blindness?	6 (30%)	47.09 ± 5.75	3.49 ± 0.82	14.83 ± 1.47
8.	Can LASIK or RK Surgery Cause Keratoconus?	5 (25%)	42.71 ± 3.89	3.21 ± 0.69	16.33 ± 2.65
9.	Are There Multiple Forms of Keratoconus?	4 (20%)	38.55 ± 4.69	2.45 ± 0.55	13.67 ± 2.16
10.	How Is Keratoconus Diagnosed?	8 (40%)	42.61 ± 2.45	3.15 ± 0.42	15.33 ± 1.50
11.	How Do You Measure the Severity of Keratoconus?	4 (20%)	43.46 ± 1.92	3.18 ± 0.42	15.5 ± 3.39
12.	How Can I Treat My Keratoconus?	16 (80%)	47.1 ± 4.33	3.55 ± 0.59	13.67 ± 1.63
13.	What is the Best Treatment for Keratoconus?	5 (25%)	46.44 ± 3.58	3.39 ± 0.53	14 ± 2.09
14.	How Can I Stop My Keratoconus From Getting Worse?	6 (30%)	44.78 ± 4.67	3.34 ± 0.52	13.67 ± 1.75
15.	Is Keratoconus Always Progressive?	9 (45%)	43.2 ± 3.35	3.1 ± 0.4	13.67 ± 1.21
16.	Does Keratoconus Cause Eye Pain?	4 (20%)	43.76 ± 4.34	2.76 ± 0.52	13.5 ± 1.51
17.	Can Keratoconus Go Away On Its Own?	3 (15%)	44.3 ± 3.56	2.85 ± 0.42	14.5 ± 1.64
18.	Can Keratoconus Cause Dry Eye?	4 (20%)	46.55 ± 3.65	3.18 ± 0.99	14.17 ± 1.72
19.	What Do I Do If I Think I Have Keratoconus?	4 (20%)	45.4 ± 3.26	3.08 ± 0.65	13.83 ± 1.72
20.	What Should Be Considered After Keratoconus Surgery?	3 (15%)	46.32 ± 2.84	3.15 ± 0.40	13.67 ± 2.06

GQS: Global Quality Score, ARLC: Average Reading Level Consensus calculator, LASIK: laser-assisted in situ keratomileusis, RK: radial keratotomy.

**Table 4:** Comparative analysis of responses from six different ai chatbots (ChatGPT-3.5, ChatGPT-4, Gemini, Copilot, Chatsonic, Perplexity) to the frequently asked questions about keratoconus: mDISCERN score, Global Quality Score (GQS), and readability indices (FRE, FKGL, ARLC).

	Chatgpt-3.5	Chatgpt-4	Gemini	Copilot	Chatsonic	Perplexity	*p*-Value
Reliability							
mDISCERN score (mean ± SD)	42.91 ± 3.15	43.1 ± 2.80	46.08 ± 5.11	46.95 ±3.53	43.97 ± 2.15	45.96 ± 3.51	<0.05
Quality							
GQS (mean ± SD)	3.02 ± 0.52	3.06 ± 0.43	3.31 ± 0.64	3.24 ± 0.55	3.10 ± 0.62	3.01 ± 0.62	0.34
Readability indexes							
FRE (mean ± SD)	21.43 ± 7.10	28.85 ± 8.44	34.7 ± 8.79	29.6 ± 9.04	24.4 ± 8.98	22.3 ± 12.75	<0.05
FKGL (mean ± SD)	15.41 ± 1.39	14.64 ± 1.70	12.46 ± 1.73	12.04 ± 1.44	13.38 ± 1.31	15.5 ± 3.02	<0.05
ARLC	15.65 ± 1.13	14.85 ± 1.53	12.9 ± 1.61	12.35 ± 1.18	13.6 ± 1.14	15.75 ± 2.55	<0.05
Response length							
Sentences (mean ± SD)	13.05 ± 4.21	13.3 ± 3.31	13 ± 5	16 ± 4.69	13.35 ± 3.78	6.9 ± 4.29	<0.05
Words (mean ± SD)	264.95 ± 78.48	285.9 ± 60.76	237.05 ± 71.85	231.3 ± 50.69	283.4 ± 70.53	127.8 ± 51.96	<0.05
Characters (mean ± SD)	1802.8 ± 538.68	1919.6 ± 425.43	1595.15 ± 489.22	1585 ± 363.31	1985.2 ± 491.80	893.6 ± 379.47	<0.05
Syllable (mean ± SD)	510.9 ± 144.98	534.6 ± 118.61	435.1 ± 129.66	442.7 ± 98.51	564.05 ± 133.18	251.6 ± 106.22	<0.05
Word/sentence (mean ± SD)	19.66 ± 2.69	20.43 ± 3.39	14.22 ± 1.96	11.3 ± 2.52	13.65 ± 2.15	16.57 ± 5.34	<0.05
Syllable/word	1.96 ± 0.06	1.89 ± 0.10	1.86 ± 0.09	1.96 ± 0.12	2.01 ± 0.10	1.99 ± 0.12	<0.05

**Table 5:** Score distribution of large language model responses according to the mDISCERN scale and quality classification. Categorical variables are presented as *n* (%).

mDISCERN Criteria	Chatgpt-3.5	Chatgpt-4	Gemini	Copilot	Chatsonic	Perplexity
	*n* = 20 (%)	*n* = 20 (%)	*n* = 20 (%)	*n* = 20 (%)	*n* = 20 (%)	*n* = 20 (%)
Excellent (63–75 points)	0 (0%)	0 (0%)	0 (0%)	0 (0%)	0 (0%)	0 (0%)
Good (51–62 points)	1 (5%)	1 (5%)	6 (30%)	4 (20%)	0 (0%)	5 (25%)
Reasonable (39–50 points)	17 (85%)	16 (80%)	12 (70%)	15 (75%)	19 (95%)	15 (75%)
Poor (27–38 points)	2 (10%)	3 (15%)	2 (10%)	1 (5%)	1 (5%)	0 (0%)
Very poor (15–26 points)	0 (0%)	0 (0%)	0 (0%)	0 (0%)	0 (0%)	0 (0%)
Quality classification						
Low quality	2 (10%)	2 (10%)	2 (10%)	1 (5%)	3 (15%)	4 (20%)
Moderate quality	15 (75%)	16 (80%)	10 (50%)	13 (65%)	11 (55%)	11 (55%)
High quality	3 (15%)	2 (10%)	8 (40%)	6 (30%)	6 (30%)	15 (25%)

## Data Availability

The original contributions presented in the study are included in the article (and supplementary material), further inquiries can be directed to the corresponding author.

## Supplementary Materials

The following supporting information can be downloaded at https://www.mdpi.com/article/10.3390/jcm13216512/s1, Table S1: Comprehensive list of web resources for frequently asked questions about keratoconus. Table S2: Responses from six different chatbots to twenty frequently asked questions about keratoconus.
