# Supplementary material for: A Performance Evaluation of Large Language Models in Keratoconus: A Comparative Study of ChatGPT-3.5, ChatGPT-4.0, Gemini, Copilot, Chatsonic, and Perplexity
Source: J Clin Med. 2024 Oct 30;13(21):6512. doi: 10.3390/jcm13216512 (PMC11547000; doi:10.3390/jcm13216512)
Supplement: Supplementary file 1 [file jcm-13-06512-s001.zip › jcm-3134695-supplementary.pdf]

Supplemental Table S1: Comprehensive List of Web Resources for Frequently Asked Questions about Keratoconus.

WEBSİTE		
**1**		https://drbleonard.com/keratoconus/keratoconus-faq/#:~:text=14.-,Does%20Keratoconus%20Cause%20Eye%20Pain%3F,thinning%2C%
**2**		https://www.insightvisionoc.com/eye-conditions/keratoconus/faqs/
**3**		https://www.allaboutvision.com/askdoc/keratoconus.htm
**4**		https://www.aao.org/eyenet/article/keratoconusquestionssolutions
**5**		https://nkcf.org/keratoconus-frequently-asked-questions/
**6**		https://www.clearvieweyes.com/keratoconus/FAQ
**7**		https://www.delmarvisionandcosmetic.com/keratoconus-faqs
**8**		https://lockfamilyeyecare.com/keratoconus-faqs
**9**		https://visioneyeinstitute.com.au/eyematters/keratoconus-faqs/
**10**		https://www.seelife.net/video-faq/faq-keratoconus/
**11**		https://www.aelc.com.au/corneal-surgery/corneal-and-keratoconus-faqs/
**12**		https://my.clevelandclinic.org/health/diseases/14415keratoconus
**13**		https://umiamihealth.org/bascom-palmer-eye-institute/specialties/corneal-and-external-diseases/keratoconus
**14**		https://wichitaoptometry.com/keratoconus-faqs
**15**		https://ct-eyecare.com/blogs/807020-keratoconus-faq
**16**		https://keratoconuscenter.eu/en/keratoconus-frequently-asked-questions/
**17**		https://www.mayoclinic.org/diseases-conditions/keratoconus/diagnosis-treatment/drc-20351357
**18**		https://www.todaysvisionconroe.com/keratoconus-faqs
**19**		https://www.yaryaneyecare.com/keratoconus-faqs
**20**		https://www.rivieraopticare.com/keratoconus-faqs

Supplemental Table S2: Responses from Six Different Chatbots to Twenty Frequently Asked Questions About Keratoconus

Question 1 :What Is Keratoconus?

ChatGPT-3.5

Keratoconus is a progressive eye condition where the cornea, which is the clear, dome-shaped surface of the eye, thins out and gradually bulges into a cone shape. This cone-like deformation causes distorted vision, sensitivity to light, and blurry eyesight because it prevents light from focusing properly on the retina.

Keratoconus often starts in the teenage years or early adulthood and can worsen over time. Early symptoms include mild blurring of vision, astigmatism, or frequent changes in prescription. As it progresses, corrective lenses might become less effective, and in severe cases, treatment options like corneal cross-linking, specialty contact lenses, or even corneal transplants may be necessary.

ChatGPT-4.0

Keratoconus is an eye condition characterized by the thinning of the cornea, which causes it to bulge outward into a cone shape, leading to distorted and blurry vision .

Symptoms of keratoconus include:

- Gradually worsening vision in one or both eyes
- Distorted vision, where straight lines may appear bent or wavy
- Increased sensitivity to light (photophobia)
- Double vision in one eye
- Halos around bright lights
- Eye redness or swelling.

Causes of keratoconus are not fully understood, but it may have a genetic component, as it can run in families. Other associated factors include eye allergies, excessive eye rubbing, and connective tissue disorders such as Marfan syndrome and Ehlers-Danlos syndrome 45.

Treatments for keratoconus vary based on the severity of the condition and may include:

- Glasses or contact lenses for mild cases
- Specialized contact lenses for more advanced cases
- Corneal cross-linking to strengthen the cornea
- In severe cases, a corneal transplant may be necessary.

Information is missing on specific treatment options and their effectiveness.

I searched for information on keratoconus to provide a comprehensive overview of its definition, symptoms, causes, and treatments, ensuring the response is accurate and informative.

Gemini

Keratoconus: A Progressive Eye Condition

Keratoconus is a progressive eye condition that causes the cornea, the clear, dome-shaped front surface of the eye, to thin and bulge outward like a cone. This abnormal shape can disrupt the way light enters the eye, leading to blurry, distorted vision.

Symptoms of Keratoconus

- Blurry vision: This is often the first symptom, especially at night or in low-light conditions.
- Double vision: This can occur as the cornea becomes more irregular.
- Light sensitivity: Difficulty tolerating bright lights or glare.
- Astigmatism: This is a common refractive error that can worsen as keratoconus progresses.
- Frequent changes in prescription: As the cornea's shape changes, your eyeglass or contact lens prescription may need frequent adjustments.

Causes of Keratoconus

The exact cause of keratoconus is unknown, but it is believed to be a combination of genetic and environmental factors. Some factors that may increase the risk include:

- Family history: If you have a family member with keratoconus, you are more likely to develop it.
- Eye rubbing: Excessive eye rubbing can weaken the cornea.
- Allergies: Allergies can contribute to eye rubbing and inflammation.
- Hormone changes: Hormonal fluctuations, such as those that occur during pregnancy or puberty, may play a role.

Treatment for Keratoconus

The treatment for keratoconus depends on the severity of the condition. Options may include:

- Contact lenses: Special contact lenses, such as rigid gas permeable (RGP) lenses or hybrid lenses, can help correct the irregular shape of the cornea.
- Corneal cross-linking (CXL): This procedure uses ultraviolet light to strengthen the cornea and slow down its progression.
- Corneal implants: In severe cases, corneal implants may be necessary to restore vision.

- Corneal transplant: In the most severe cases, a corneal transplant may be required to replace the damaged cornea with a healthy donor cornea.

If you are experiencing any of the symptoms of keratoconus, it's important to see an eye doctor

Copilot

Keratoconus is an eye condition where the cornea, the clear, dome-shaped front surface of the eye, becomes thinner and gradually bulges outward into a cone shape . This change in shape causes light entering the eye to be deflected, leading to distorted and blurry vision .

Symptoms

- Blurred or distorted vision
- Increased sensitivity to light and glare
- Frequent changes in eyeglass prescriptions
- Difficulty seeing at night
- Eye redness or swelling

Causes

The exact cause of keratoconus is unknown, but it is believed to be a combination of genetic, environmental, and hormonal factors. It often starts in the late teens to early 20s and can progress over time .

Treatment

Treatment options depend on the severity of the condition:

- **Eyeglasses or soft contact lenses** for mild cases
- **Rigid gas permeable contact lenses** for more advanced cases
- **Corneal cross-linking** to strengthen the cornea
- **Intacs** (small curved devices inserted into the cornea)
- **Corneal transplant** in severe cases

If you have any concerns about your vision, it's important to consult an eye care professional. Do you have any specific questions about keratoconus or eye health in general?

Chatsonic

Here's a comprehensive overview of keratoconus, structured to enhance readability and understanding:

1. Definition

Keratoconus is a progressive eye disorder where the normally round, dome-shaped cornea thins and begins to bulge into a cone-like shape. This distortion of the cornea leads to vision problems because it affects how light enters the eye and is focused on the retina.

2. Causes

The exact cause of keratoconus is not fully understood, but several factors are believed to contribute to its development:

- **Genetic Factors**: A family history of keratoconus increases the risk, suggesting a genetic component.
- **Environmental Factors**: Frequent eye rubbing, allergies, and exposure to ultraviolet rays are considered potential risk factors.
- **Biochemical Factors**: Imbalances in the cornea's enzyme activity can lead to weakening and thinning of the corneal tissue.

3. Symptoms

Common symptoms of keratoconus include:

- Blurred or distorted vision
- Increased sensitivity to light and glare
- Frequent changes in eyeglass prescriptions
- Difficulty seeing at night
- Eye strain or headaches, especially after reading or using a computer

4. Diagnosis

Healthcare professionals diagnose keratoconus through several methods:

- **Eye Examination**: A comprehensive eye exam to check for vision changes.
- **Corneal Topography**: A specialized imaging technique that maps the curvature of the cornea, identifying any irregularities.
- **Pachymetry**: Measures the thickness of the cornea to detect thinning.
- **Slit-Lamp Examination**: Allows detailed examination of the cornea's structure.

5. Treatment Options

Treatment for keratoconus varies depending on the severity of the condition:

Non-Surgical Approaches

- **Glasses or Soft Contact Lenses**: For mild cases, to correct vision.
- **Rigid Gas Permeable (RGP) Contact Lenses**: Provide a more stable surface to improve vision.
- **Scleral Lenses**: Larger lenses that vault over the cornea, offering comfort and improved vision.

Surgical Approaches

- **Corneal Cross-Linking**: A minimally invasive procedure that strengthens corneal fibers to halt progression.
- **Intacs**: Small, arc-shaped inserts placed in the cornea to flatten it and improve vision.
- **Corneal Transplant**: In severe cases, a damaged cornea may be replaced with a donor cornea.

6. Prognosis

The long-term outlook for individuals with keratoconus varies:

- **Early Detection and Treatment**: Can significantly slow progression and improve quality of life.
- **Stable Vision**: Many individuals achieve stable vision with appropriate treatment.
- **Advanced Cases**: May require surgical intervention, but outcomes are generally positive with modern techniques.

Keratoconus is a manageable condition with proper medical care and advances in treatment options, allowing many individuals to maintain good vision and lead normal lives.

Perplexity

Keratoconus is a progressive eye condition characterized by a thinning and bulging of the cornea, the clear front surface of the eye, causing it to take on a cone-like shape. This abnormal corneal shape leads to distorted vision, including nearsightedness, astigmatism, and irregular astigmatism.

Key Points About Keratoconus

- It typically begins in adolescence or early adulthood and can progress slowly over several years.
- In severe cases, the cornea may become scarred, leading to further vision loss.
- Keratoconus usually affects both eyes, although one eye may be more severely affected than the other.
- The exact underlying cause is not fully understood, but it is believed to be caused by a combination of genetic and environmental factors.

Symptoms

The symptoms of keratoconus can vary from person to person and may include:

- Blurred or distorted vision
- Increased sensitivity to bright light and glare
- Frequent changes in eyeglass prescriptions
- Sudden worsening or clouding of vision
- Glare and halos around lights
- Difficulty seeing at night
- Eye irritation or headaches associated with eye pain

Diagnosis and Treatment

Keratoconus is typically diagnosed through a comprehensive eye exam, which may include:

- Visual acuity test
- Slit-lamp exam to detect abnormalities in the cornea
- Corneal topography to create a detailed map of the cornea's shape
- Pachymetry to measure corneal thickness

Treatment options depend on the stage of the disease and may include:

- Glasses or soft contact lenses in early stages
- Rigid, gas permeable contact lenses or scleral lenses as the disease progresses
- Corneal collagen cross-linking to slow or stop the progression of the disease
- Corneal transplant surgery in advanced cases

Regular eye exams and adherence to prescribed treatments are important to manage keratoconus and minimize its impact on vision and quality of life.

Question 2: What Are the Symptoms of Keratoconus?

ChatGPT-3.5

The symptoms of keratoconus typically progress as the condition worsens. Common symptoms include:

1. Blurred or distorted vision – due to the irregular shape of the cornea.
2. Increased sensitivity to light and glare – especially when driving at night.
3. Frequent changes in eyeglass prescriptions – as the cornea continues to change shape.
4. Double vision or "ghosting" – objects may appear doubled or have halos around them.
5. Worsening nearsightedness or astigmatism – as the condition progresses.
6. Difficulty seeing clearly at night – night vision tends to deteriorate.
7. Eye irritation or discomfort – some people may experience a gritty feeling or mild irritation.

If left untreated, keratoconus can lead to severe vision problems.

ChatGPT-4.0

I will extract and summarize the symptoms of keratoconus from the search results obtained earlier to provide a clear and concise list. The symptoms of keratoconus include:

- Gradually worsening vision in one or both eyes
- Distorted vision, where straight lines may appear bent or wavy
- Increased sensitivity to light (photophobia)
- Double vision in one eye
- Halos around bright lights
- Eye redness or swelling

These symptoms can vary in severity and may progress over time.

Gemini

Keratoconus is a progressive eye condition that causes the cornea, the clear, dome-shaped front surface of the eye, to thin and bulge outward like a cone. This abnormal shape can disrupt the way light enters the eye, leading to blurry, distorted vision.

Common symptoms of keratoconus include:

- **Blurry vision:** This is often the first symptom, especially at night or in low-light conditions.
- **Double vision:** This can occur as the cornea becomes more irregular.
- **Light sensitivity:** Difficulty tolerating bright lights or glare.
- **Astigmatism:** This is a common refractive error that can worsen as keratoconus progresses.
- **Frequent changes in prescription:** As the cornea's shape changes, your eyeglass or contact lens prescription may need frequent adjustments.

If you are experiencing any of these symptoms, it's important to see an eye doctor for a comprehensive eye exam. Early diagnosis and treatment can help prevent vision loss.

Copilot

The symptoms of keratoconus can vary as the condition progresses. Here are some common signs to look out for:

Early Symptoms

- **Blurred or distorted vision**: This is often the first noticeable symptom.
- **Increased sensitivity to light and glare**: Bright lights may cause discomfort.
- **Frequent changes in eyeglass prescriptions**: You might need new prescriptions more often than usual.
- **Difficulty seeing at night**: Night vision can become particularly challenging.

Advanced Symptoms

- **Double vision**: Seeing two images instead of one when looking with one eye.
- **Seeing halos around bright lights**: Lights may appear to have rings around them.
- **Worsening vision**: Vision may continue to deteriorate over time.
- **Eye redness or swelling**: The eyes may become irritated and swollen[123].

If you notice any of these symptoms, it's important to consult an eye care professional for a proper diagnosis and treatment plan. Do you have any other questions about keratoconus or eye health?

Chatsonic

The symptoms of keratoconus can vary depending on the severity of the condition but typically include:

- **Blurred or Distorted Vision**: As the cornea changes shape, vision becomes blurry or distorted.
- **Increased Sensitivity to Light and Glare**: Bright lights may cause discomfort or halos.
- **Frequent Changes in Eyeglass Prescriptions**: Vision can fluctuate, requiring frequent updates to prescriptions.
- **Difficulty Seeing at Night**: Night vision may be particularly challenging.
- **Eye Strain or Headaches**: Especially after activities like reading or using a computer.
- **Double Vision in One Eye**: Known as monocular diplopia, this can occur in more advanced cases.

These symptoms often start in the late teens to early twenties and can progress over time.

Perplexity

The symptoms of keratoconus can vary among individuals and may change as the condition progresses. Common symptoms include:

- **Mild Blurring of Vision**: The earliest sign is often a slight blurring or progressively poor vision that isn't easily corrected with glasses.

- **Distorted Vision**: Straight lines may appear bent or wavy, leading to difficulties in tasks requiring clear vision.
- **Increased Sensitivity to Light**: Patients often experience heightened sensitivity to bright lights and glare.
- **Glare and Halos**: Many individuals report seeing glare and halos around lights, especially at night.
- **Difficulty Seeing at Night**: Night vision may become particularly challenging.
- **Eye Irritation or Pain**: Some may experience discomfort, which can be accompanied by headaches.
- **Frequent Changes in Prescription**: Individuals may notice they need to change their eyeglass or contact lens prescriptions more frequently as their vision deteriorates.
- **Sudden Worsening of Vision**: There may be instances of rapid decline in vision quality, including clouding.

If you experience any of these symptoms, it is important to consult an eye care professional for a comprehensive evaluation.

Question 3: How Do Patients with Keratoconus See ?

ChatGPT-3.5

Patients with keratoconus often experience distorted and blurry vision due to the irregular cone-shaped cornea. Their vision is usually characterized by:

1. **Blurred or distorted images** – Objects appear wavy or stretched, and straight lines might look bent or warped.
2. **Double vision or ghosting** – Multiple images of a single object may appear, especially in one eye, causing a "ghosting" effect.
3. **Halos and glare** – Around lights, particularly at night, halos, glare, or starbursts may be noticeable.
4. **Difficulty with night vision** – Low-light situations, like driving at night, can be particularly challenging.
5. **Frequent prescription changes** – Glasses or contact lenses often need adjustment as the cornea's shape changes.

ChatGPT-4.0

Keratoconus significantly impacts visual perception due to the progressive thinning and irregular bulging of the cornea, which leads to irregular astigmatism and myopia. This condition typically begins in puberty and can result in blurred vision, difficulty seeing at night, and visual distortions such as glare and halos around

lights . As the disease progresses, patients may experience increased higher-order aberrations, further impairing visual clarity and quality .<br><br>Visual acuity is often affected differently in each eye, with the "better" eye being crucial for overall vision-related quality of life (VRQoL) . Patients may also report a reduced ability to engage in activities that require clear vision, such as reading or enjoying visual experiences, which can lead to emotional and social challenges . Overall, keratoconus can lead to significant visual impairment, affecting daily activities and quality of life.<br><br>.
**Gemini**
**Patients with keratoconus often experience blurry, distorted vision.** This is because the cone-shaped cornea interferes with the way light enters the eye. The vision may be especially blurry at night or in low-light conditions.<br><br>**Other visual symptoms of keratoconus may include:**<br><br>• **Double vision:** The irregular shape of the cornea can cause objects to appear double.<br>• **Light sensitivity:** Patients with keratoconus may find it difficult to tolerate bright lights or glare.<br>• **Astigmatism:** This is a common refractive error that can worsen as keratoconus progresses.<br><br>**It's important to note that the severity of vision problems can vary greatly from person to person, depending on the stage of keratoconus.** Some people may experience only mild symptoms, while others may have more severe vision loss.
**Copilot**
Patients with keratoconus often experience distorted and blurry vision due to the irregular shape of their cornea. Here are some common visual experiences they might have:<br><br>**Visual Distortions**<br><br>• **Blurred Vision**: Objects may appear out of focus or smeared.<br>• **Double Vision**: Seeing two images of a single object, especially when looking with one eye.<br>• **Halos and Glare**: Bright lights may have rings around them, and glare can be more pronounced.<br>• **Ghosting**: Multiple images or “ghost” images of a single object, particularly noticeable at night.<br><br>**Impact on Daily Life**<br><br>• **Difficulty Reading**: Small print can be hard to read, and letters may appear to overlap.<br>• **Challenges with Night Vision**: Driving at night can be particularly difficult due to increased glare and halos around lights.

- **Frequent Prescription Changes**: Glasses or contact lenses may need to be updated more often as the condition progresses.

Visual Aids

- **Specialized Contact Lenses**: Rigid gas permeable lenses or scleral lenses can help provide clearer vision by creating a smooth surface over the irregular cornea.
- **Eyeglasses**: In early stages, glasses may help, but they often become less effective as keratoconus progresses.

If you or someone you know is experiencing these symptoms, it's important to consult an eye care professional for proper diagnosis and treatment. Do you have any other questions about keratoconus or vision health?

Chatsonic

Patients with keratoconus experience vision that is often described as distorted or blurry due to the irregular shape of the cornea. Here's how their vision might be affected:

- **Blurred Vision**: Objects may appear out of focus, similar to looking through a foggy or smeared glass.
- **Distorted Vision**: Straight lines can appear wavy or bent, and shapes may seem stretched or compressed.
- **Multiple Images**: Some patients report seeing multiple images of a single object, a phenomenon known as ghosting or double vision in one eye (monocular diplopia).
- **Halos and Glare**: Bright lights can create halos or starburst patterns, making it difficult to see in bright conditions or at night.
- **Increased Sensitivity to Light**: Patients may find bright lights uncomfortable or overwhelming.

These visual disturbances result from the cornea's uneven surface, which disrupts the way light is focused onto the retina.

Perplexity

Patients with keratoconus experience significant visual disturbances due to the progressive thinning and bulging of the cornea into a cone shape. This abnormal corneal shape leads to various vision-related issues, including:

- **Blurry Vision**: The irregular shape of the cornea causes light to enter the eye unevenly, resulting in blurred vision that may not be fully correctable with glasses or standard contact lenses.

- **Distorted Vision**: Patients often see straight lines as wavy or bent, which can complicate tasks such as reading or driving.
- **Increased Sensitivity to Light**: Many individuals with keratoconus report heightened sensitivity to bright lights and glare, which can make it difficult to see in well-lit environments or at night.
- **Halos Around Lights**: Patients frequently notice halos or glare around lights, particularly in low-light conditions, affecting night vision.
- **Frequent Changes in Prescription**: As the condition progresses, individuals may need to update their eyeglass or contact lens prescriptions more often due to rapid changes in vision.
- **Double Vision**: Some patients experience double vision when looking with one eye, which can further impair their ability to see clearly.

Overall, the visual experience of patients with keratoconus can be quite challenging, impacting daily activities and overall quality of life. Regular eye examinations and appropriate vision correction methods are essential for managing the condition effectively.

Question 4: How Can Keratoconus Affect My Life?

ChatGPT-3.5

Keratoconus can have a significant impact on daily life, particularly as the condition progresses. Here are some ways it might affect you:

1. **Visual Impairment**: The distorted vision can make reading, driving, especially at night, and recognizing faces more difficult. These challenges can be frustrating and affect your independence.
2. **Frequent Prescription Changes**: You may need to update your glasses or contact lenses frequently as the shape of your cornea changes, adding inconvenience and cost.
3. **Work and Education**: Jobs or academic tasks that require fine visual detail, like reading or working with computers, may become harder, affecting performance.
4. **Physical Activities**: Sports and activities requiring good vision or hand-eye coordination may be more challenging. Also, eye protection becomes important to prevent trauma to the cornea.
5. **Emotional and Social Impact**: Living with impaired vision can lead to feelings of frustration, anxiety, or self-consciousness, especially in social or work situations.

Treatment, such as specialty contact lenses or corneal procedures, can help manage the condition and mitigate its impact on your life.

ChatGPT-4.0

Keratoconus can affect various aspects of your life due to its impact on vision. Here's an overview of how it might influence different areas:

1. Daily activities:
   - Reading, watching TV, or using digital devices may become challenging
   - Driving, especially at night, can be difficult and potentially unsafe
   - Recognizing faces from a distance might be harder
2. Education and work:
   - Reading, writing, and computer work may require additional effort or accommodations
   - Certain careers that require excellent vision might be impacted
3. Sports and hobbies:
   - Activities requiring good depth perception or visual acuity may become more challenging
   - Some contact sports might need to be avoided to prevent eye injury
4. Emotional well-being:
   - Frustration or anxiety due to vision changes
   - Potential impact on self-esteem or confidence
5. Social interactions:
   - Difficulty recognizing people or reading social cues from a distance
   - Explaining the condition to others who may not understand the visual challenges
6. Financial considerations:
   - Costs associated with treatments, specialized contact lenses, or surgeries
   - Potential impact on career choices or job performance
7. Medical management:
   - Regular eye exams and monitoring of the condition
   - Adapting to different vision correction methods as the condition progresses
8. Lifestyle adjustments:
   - Learning to manage glare and light sensitivity
   - Developing strategies to cope with vision changes in various environments

It's important to note that the impact of keratoconus can vary greatly from person to person, depending on the severity of the condition and how it progresses. Many people with keratoconus lead full, active lives with proper management and treatment. Working closely with an eye care professional can help in developing strategies to minimize the impact on your daily life and maintain good quality of life.

Gemini
**Keratoconus can significantly impact your daily life, especially if it progresses.** The condition can affect your ability to:<br><br>• **Perform tasks that require clear vision:** Reading, driving, working on a computer, and participating in hobbies or sports can become challenging.<br>• **Maintain independence:** Difficulty with vision can limit your ability to perform tasks independently, such as getting dressed, cooking, or navigating your surroundings.<br>• **Socialize and interact with others:** Vision problems can affect your confidence and self-esteem, making it more difficult to socialize and participate in social activities.<br>• **Work and study:** If your vision is significantly impaired, it may be difficult to maintain your job or pursue your education.<br><br>**However, with appropriate treatment and management, you can still lead a fulfilling life.** Advances in medical technology have made it possible for many people with keratoconus to maintain good vision and live relatively normal lives.<br><br>It's important to **work closely with your eye doctor** to develop a treatment plan that addresses your specific needs and helps you manage the impact of keratoconus on your life.
**Copilot**
Keratoconus can have a significant impact on various aspects of your life, especially if it progresses. Here are some ways it might affect you:<br><br>**Daily Activities**<br><br>• **Reading and Writing**: Blurred or distorted vision can make it difficult to read books, screens, or even handwritten notes.<br>• **Driving**: Night driving can become particularly challenging due to increased glare and halos around lights.<br>• **Work and School**: Tasks that require clear vision, such as working on a computer or reading, can become more difficult and may require frequent breaks or adjustments.<br><br>**Emotional and Social Impact**<br><br>• **Frustration and Stress**: Frequent changes in vision and the need for new prescriptions can be frustrating.<br>• **Social Interactions**: Difficulty seeing clearly can affect your confidence in social settings, especially in low-light conditions.

- **Mental Health**: The ongoing challenges with vision can lead to anxiety or depression in some individuals.

Physical Discomfort

- **Eye Strain**: Constantly trying to focus can lead to eye strain and headaches.
- **Discomfort with Contact Lenses**: Some people may find rigid gas permeable lenses uncomfortable, although they are often necessary for better vision.

Adaptations and Solutions

- **Specialized Eyewear**: Using rigid gas permeable or scleral lenses can significantly improve vision.
- **Assistive Devices**: Magnifiers, screen readers, and other assistive technologies can help with reading and other tasks.
- **Regular Eye Check-ups**: Frequent visits to an eye care professional can help manage the condition and adjust treatments as needed.

Positive Outlook

- **Treatment Advances**: Modern treatments like corneal cross-linking can help stabilize the condition and prevent further deterioration.
- **Support Systems**: Joining support groups or talking to others with keratoconus can provide emotional support and practical advice.

If you have any specific concerns or need advice on managing keratoconus, feel free to ask. Your eye care professional can also provide personalized guidance and treatment options.

Chatsonic

Keratoconus can have several impacts on daily life, depending on the severity of the condition. Here are some ways it might affect you:

1. Vision Challenges

- **Daily Activities**: Tasks that require clear vision, such as reading, driving, or using a computer, can become more difficult.
- **Night Vision**: Driving at night or in low-light conditions may be particularly challenging due to glare and halos around lights.

2. Emotional and Psychological Impact

- **Stress and Anxiety**: The progressive nature of keratoconus can cause worry about future vision loss.
- **Frustration**: Frequent changes in vision and corrective lenses can be frustrating and inconvenient.

3. Financial Considerations

- **Cost of Treatment**: Expenses related to frequent eye exams, glasses, contact lenses, or surgical procedures can add up.
- **Time Off Work**: Appointments and recovery from treatments may require time away from work or other responsibilities.

4. Lifestyle Adjustments

- **Adaptations**: You may need to make adjustments to your environment, such as improving lighting or using magnifying aids for reading.
- **Activity Limitations**: Certain activities, especially those requiring sharp vision, might be limited.

5. Social and Professional Life

- **Driving Restrictions**: If vision is significantly impaired, driving may become unsafe, affecting independence and mobility.
- **Job Performance**: Jobs that rely heavily on visual tasks might require accommodations or adjustments.

With proper management and treatment, many individuals with keratoconus can lead full and active lives. Early detection and regular monitoring are key to minimizing its impact.

Perplexity

Keratoconus can significantly impact various aspects of your daily life, including:

Vision and Activities

- **Blurred and distorted vision** can make it difficult to perform tasks like watching TV, reading, or using digital screens.
- **Increased sensitivity to light and glare**, especially at night, can impair driving and outdoor activities.
- **Frequent changes in eyeglass or contact lens prescriptions** may be needed as the condition progresses.

Work and Career

- **Keratoconus can lead to career setbacks**, such as forced changes or job loss due to vision impairment.
- **Patients may experience financial hardships** due to the cost of treatments and vision aids.

Social and Personal Life

- **Keratoconus can strain personal relationships** and lead to feelings of isolation and discrimination.
- **Enjoyment of hobbies and normal life activities** may be limited by the condition.

Emotional Well-being

- **Patients often experience frustration with treatment effectiveness** and fear of disease progression.
- **Emotional strain and anxiety** are common due to the chronic nature of the condition.

To cope with the impact of keratoconus:

- **Seek early treatment** to stabilize vision and prevent further deterioration
- **Communicate with eye care professionals** about changes in symptoms
- **Use artificial tears and avoid eye rubbing** to manage dry eyes
- **Adjust daily activities** to accommodate vision challenges
- **Seek support from family, friends, and support groups** to manage emotional well-being

Regular eye exams and adherence to prescribed treatments are crucial to managing keratoconus and minimizing its impact on quality of life. Seeking help from eye care professionals and support networks can also help individuals cope with the challenges posed by this condition.

Question 5: How Common Is Keratoconus?

ChatGPT-3.5

Keratoconus is relatively uncommon, affecting about 1 in 2,000 people worldwide. However, some studies suggest it may be more prevalent, as mild cases might go undiagnosed. It typically begins in adolescence or early adulthood and can progress for 10-20 years before stabilizing.

Certain factors, such as a family history of keratoconus, frequent eye rubbing, or conditions like Down syndrome or asthma, can increase the risk of developing the condition.

ChatGPT-4.0

Keratoconus is relatively uncommon, with an estimated prevalence of about 1 in 2,000 people worldwide. This estimate is based on epidemiological studies, though the prevalence can vary depending on geographic and demographic factors.

Gemini

Keratoconus is a relatively uncommon eye condition. While the exact prevalence varies depending on population and geographic location, it is generally estimated to affect between 1 in 2,000 to 1 in 250 people.

Some factors that may contribute to the prevalence of keratoconus include:

- **Genetics:** A family history of keratoconus can increase your risk.
- **Eye rubbing:** Excessive eye rubbing can weaken the cornea and contribute to the development of keratoconus.
- **Allergies:** Allergies can lead to eye rubbing and inflammation.
- **Hormone changes:** Hormonal fluctuations, such as those that occur during pregnancy or puberty, may play a role.

If you are concerned about your risk of developing keratoconus, it's important to **see an eye doctor for regular eye exams.** Early diagnosis and treatment can help prevent vision loss.

Copilot

Keratoconus is relatively uncommon but not rare. The global prevalence is estimated to be around 1.4 per 1,000 people1. However, this can vary significantly by region. For example, in some areas, the prevalence can be as high as 40 per 1,0001.

It's more frequently diagnosed in regions like the Middle East, India, China, and Australia2. The condition affects both men and women equally and is often diagnosed in the second or third decade of life3.

Chatsonic

The prevalence of keratoconus has been found to be higher than previously thought, with recent studies indicating significant variability based on geographical and demographic factors. While earlier estimates suggested it affected about 1 in 2,000 people, newer research shows that the prevalence can be as high as 1 in 84 in certain populations, such as young adults in Australia 1. Other studies have reported prevalence rates ranging from 1 in 1,000 in Israeli adolescents to as high as 1 in 20 among Saudi Arabian children 3. This variation is influenced by factors like ethnicity and the diagnostic methods used in different studies.

Perplexity

The prevalence of keratoconus varies significantly across different populations and geographic regions. Key findings regarding its prevalence include:

- **Global Estimates**: The global prevalence of keratoconus has been reported to be approximately 1.4 per 1,000 individuals, which translates to about 0.14% of the population.
- **Regional Variations**:
  - In Central India, prevalence rates can be as high as 2.3% (2300 per 100,000) based on specific diagnostic criteria.

- In contrast, lower prevalence rates have been noted in regions such as Russia, where the rate is approximately 0.3 per 100,000.
- A study in the USA reported a prevalence of about 0.054% in Minnesota, while other studies indicated rates of 0.19% in Norway and 0.51% in Colorado.

- **Ethnic and Environmental Factors**: The prevalence of keratoconus tends to be higher in certain ethnic groups, particularly among Arabs and Asians, compared to Caucasians. For instance, studies have shown that the prevalence is significantly higher in Asian populations in the UK, with rates being 4.4 to 7.5 times greater than those in white Caucasians.
- **Age Considerations**: Keratoconus commonly manifests during late adolescence to early adulthood, and its prevalence may be underestimated in older populations due to the progressive nature of the disease.

Overall, while keratoconus is often considered a rare condition, its prevalence can be much higher in specific groups and regions, highlighting the importance of targeted screening and awareness in populations at risk.

Question 6: What Causes Keratoconus?

ChatGPT-3.5

The exact cause of keratoconus is still unknown, but several factors are thought to contribute to its development:

1. **Genetic Factors**: Keratoconus often runs in families, suggesting a genetic link. If a close relative has it, you may be at higher risk.
2. **Eye Rubbing**: Frequent or vigorous eye rubbing is associated with a higher risk of developing keratoconus, especially in people with conditions like allergies.
3. **Environmental Factors**: Exposure to UV light, poorly fitted contact lenses, or chronic eye irritation may play a role.
4. **Underlying Conditions**: It is more common in people with certain medical conditions, such as Down syndrome, asthma, sleep apnea, and connective tissue disorders like Marfan or Ehlers-Danlos syndromes.

Keratoconus is likely caused by a combination of genetic and environmental factors that lead to the thinning and weakening of the cornea over time.

ChatGPT-4.0

Keratoconus is a complex condition with multiple contributing factors:

1. **Genetic Factors**:

- Family history: Individuals with a family history of keratoconus are at a higher risk, suggesting a genetic predisposition.
- Genetic mutations: Research has identified several genetic mutations associated with keratoconus, though the exact genetic mechanisms are not fully understood.

2. **Environmental Factors**:

- Eye rubbing: Frequent and vigorous eye rubbing is a known risk factor, potentially exacerbating the thinning and weakening of the cornea.
- Allergies: Conditions like hay fever and asthma, which cause eye itching and rubbing, are associated with keratoconus.
- Ultraviolet (UV) exposure: Prolonged exposure to UV rays may contribute to corneal damage and the progression of keratoconus.

3. **Biochemical Factors**:

- Corneal structure: The cornea's collagen fibers provide structural support. In keratoconus, these fibers are weakened, leading to corneal thinning and bulging.
- Enzymatic activity: Imbalances in enzymes that regulate collagen and other corneal components may contribute to the degradation of corneal tissue.

4. **Systemic Conditions**:

- Certain systemic conditions, such as Down syndrome, Ehlers-Danlos syndrome, and Marfan syndrome, have been linked to a higher incidence of keratoconus.

5. **Inflammatory Processes**:

- Chronic inflammation of the cornea may play a role in the development and progression of keratoconus.

Gemini

The exact cause of keratoconus remains unknown. However, it is believed to be a combination of genetic and environmental factors.

Here are some factors that may increase your risk of developing keratoconus:

- **Genetics:** If you have a family history of keratoconus, you are more likely to develop it.
- **Eye rubbing:** Excessive eye rubbing can weaken the cornea.
- **Allergies:** Allergies can contribute to eye rubbing and inflammation.
- **Hormone changes:** Hormonal fluctuations, such as those that occur during pregnancy or puberty, may play a role.
- **Connective tissue disorders:** Conditions like Marfan syndrome and Ehlers-Danlos syndrome can increase the risk of keratoconus.

While these factors may increase your risk, it's important to note that not everyone who has these factors will develop keratoconus.
**Copilot**
The exact cause of keratoconus isn't fully understood, but it's believed to result from a combination of genetic, environmental, and possibly hormonal factors[1][2].<br><br>Here are some key factors associated with keratoconus:<br><br>1. **Genetics**: About 1 in 10 people with keratoconus have a family member who also has it[2].<br>2. **Eye Rubbing**: Frequent and vigorous eye rubbing is linked to the development and progression of keratoconus[2].<br>3. **Allergies**: Conditions like hay fever, asthma, and eczema are often associated with keratoconus[2].<br>4. **Connective Tissue Disorders**: Conditions such as Marfan syndrome and Ehlers-Danlos syndrome can be linked to keratoconus[2].
**Chatsonic**
The exact cause of keratoconus is not fully understood, but it is believed to result from a combination of genetic, environmental, and cellular factors. Here are some key contributors:<br><br>**1. Genetic Factors**<br><br>• **Family History**: Keratoconus often runs in families, suggesting a genetic predisposition. If a close family member has the condition, the risk may be higher.<br>• **Genetic Mutations**: Certain genetic mutations have been associated with keratoconus, although the specific genes involved are still being studied.<br><br>**2. Environmental Factors**<br><br>• **Eye Rubbing**: Frequent or vigorous rubbing of the eyes is considered a significant risk factor. It can weaken the corneal tissue over time.<br>• **Allergies**: Conditions that cause itching and lead to eye rubbing, such as hay fever, may contribute to the development of keratoconus.<br><br>**3. Cellular and Biochemical Factors**<br><br>• **Corneal Structure**: The cornea's collagen fibers may be weaker or less organized in individuals with keratoconus, leading to its thinning and bulging.<br>• **Enzymatic Activity**: Imbalances in enzymes that protect the cornea from oxidative damage may play a role in the disease's progression.<br><br>**4. Systemic Conditions**

- **Associated Disorders**: Keratoconus is sometimes associated with systemic conditions like Down syndrome, Marfan syndrome, and Ehlers-Danlos syndrome.

Understanding these factors can help in managing and potentially preventing the progression of keratoconus. Regular eye check-ups are important for early detection and treatment.

Perplexity

Keratoconus is a complex eye condition, and while its exact cause remains unclear, several factors are believed to contribute to its development. Key causes and risk factors include:

Genetic Factors

- **Family History**: Approximately 10% of individuals with keratoconus have a family member with the condition, suggesting a genetic predisposition. This indicates that certain genetic factors may increase the likelihood of developing keratoconus, although it is not strictly inherited in all cases.

Environmental Factors

- **Eye Rubbing**: Chronic rubbing of the eyes is associated with keratoconus and may exacerbate the condition. This behavior can lead to mechanical trauma to the cornea, contributing to its thinning and irregular shape.
- **Ultraviolet Light Exposure**: Prolonged exposure to UV rays may play a role in the development of keratoconus, although the exact mechanism is not fully understood.
- **Chronic Eye Irritation**: Conditions that cause persistent inflammation or irritation, such as allergies, can contribute to the weakening of corneal tissue and the progression of keratoconus.

Other Contributing Factors

- **Collagen Deficiency**: Keratoconus is characterized by a loss of collagen in the cornea, which can lead to its weakening and bulging. An imbalance between the production and breakdown of collagen may be a significant factor in the disease's progression.
- **Age**: The condition typically begins in late adolescence or early adulthood and may progress into the mid-30s. The progression rate can vary significantly among individuals.

While the precise mechanisms behind keratoconus are still under investigation, understanding these contributing factors can help in managing the condition and potentially guiding preventive measures. Regular eye examinations and early intervention are crucial for those at risk.

Question 7: Does Keratoconus Cause Blindness?

ChatGPT-3.5

Keratoconus does not typically cause complete blindness, but it can lead to severe vision impairment if left untreated. As the cornea thins and bulges, vision becomes increasingly distorted, making it difficult to perform daily tasks like reading or driving.<br><br>In severe cases, the cornea may scar or swell, significantly reducing vision quality. While this can lead to functional blindness (where normal vision is no longer possible), modern treatments such as specialty contact lenses, corneal cross-linking, and, in advanced cases, corneal transplants can help prevent serious vision loss and improve quality of life.
**ChatGPT-4.0**
Keratoconus does not cause complete blindness, but it can lead to significant visual impairment. The condition causes the cornea to thin and bulge into a cone shape, leading to distorted and blurred vision. This can severely affect daily activities such as reading, driving, and recognizing faces. However, with appropriate management and treatment, the progression of keratoconus can be controlled, and vision can be improved.
**Gemini**
**No, keratoconus does not typically cause complete blindness.** While it can lead to significant vision loss, there are effective treatment options available to help manage the condition and prevent total blindness.<br><br>**However, if left untreated, keratoconus can progress to a point where it becomes difficult to correct with contact lenses or eyeglasses.** In severe cases, a corneal transplant may be necessary to restore vision.<br><br>It's important to **see an eye doctor regularly** to monitor the progression of keratoconus and receive appropriate treatment. Early diagnosis and intervention can help prevent vision loss and maintain a good quality of life.
**Copilot**
Keratoconus typically does not cause complete blindness, but it can significantly impair vision if left untreated. The condition causes the cornea to thin and bulge into a cone-like shape, leading to distorted and blurred vision. In severe cases, it can result in scarring of the cornea, which further reduces vision quality.<br><br>However, with proper management and treatment, most people with keratoconus can maintain good vision. Treatments include:<br><br>• **Glasses or Contact Lenses**: In the early stages, glasses or soft contact lenses can correct vision.<br>• **Rigid Gas Permeable (RGP) Lenses**: These are often used when glasses or soft lenses no longer provide adequate vision correction.<br>• **Corneal Cross-Linking**: A procedure that strengthens the cornea to prevent further bulging.

- **Corneal Transplant**: In advanced cases, a corneal transplant may be necessary.

Chatsonic

Keratoconus does not typically cause complete blindness, but it can lead to significant vision impairment if left untreated. Here's how it can affect vision:

Vision Impairment

- **Distorted Vision**: The irregular shape of the cornea causes light to be refracted improperly, leading to blurred and distorted vision.
- **Progressive Condition**: As keratoconus progresses, the cornea continues to thin and bulge, worsening the visual distortions.
- **Severe Cases**: In advanced stages, scarring of the cornea can occur, further reducing vision quality.

Management and Treatment

- **Corrective Lenses**: Glasses or contact lenses can often correct vision in the early stages.
- **Surgical Options**: Procedures like corneal cross-linking, intacs, or corneal transplants can help stabilize or improve vision in more advanced cases.

Prevention of Severe Outcomes

- **Regular Monitoring**: Early detection and regular monitoring by an eye care professional can help manage the condition effectively and prevent severe vision loss.

While keratoconus can significantly impact vision, with proper management, most people can maintain functional vision and lead normal lives.

Perplexity

Keratoconus can lead to significant visual impairment, but it does not typically cause complete blindness. The condition is characterized by the thinning and bulging of the cornea, which can result in distorted and blurred vision. However, most patients retain some level of vision, especially in the early stages of the disease.

Key Points Regarding Vision Loss in Keratoconus:

- **Progressive Nature**: Keratoconus is a progressive condition that usually begins in adolescence and can worsen over time. As it advances, the cornea may become more irregular, leading to increased visual distortion and difficulty seeing clearly.
- **Visual Impairment**: In moderate to advanced stages, keratoconus can significantly affect visual acuity, often requiring specialized contact lenses or surgical interventions such as corneal

transplantation to restore vision. However, the frequency of severe visual impairment in keratoconus patients is low, and complete blindness is rare.

- **Treatment Options**: Early diagnosis and treatment are crucial. Options such as rigid gas permeable contact lenses, corneal collagen cross-linking, and corneal transplants can help manage the condition and preserve vision. Studies indicate that with appropriate treatment, the risk of severe visual impairment is minimized.

In summary, while keratoconus can lead to significant vision challenges, it does not typically result in total blindness, especially with timely and effective management. Regular eye examinations and adherence to treatment plans are essential for maintaining vision quality.

Question 8: Can LASIK or RK Surgery Cause Keratoconus?

ChatGPT-3.5

Yes, LASIK or RK (Radial Keratotomy) surgeries can potentially cause a condition similar to keratoconus known as **post-LASIK ectasia** or **post-surgical ectasia**. These refractive surgeries reshape the cornea to correct vision, and in some cases, they can weaken the corneal structure, leading to a progressive thinning and bulging, similar to keratoconus.

This risk is higher if the cornea was already thin or had irregularities before surgery, which is why thorough preoperative screening is crucial to avoid complications like ectasia. If you are at risk of keratoconus or have a thin cornea, doctors may recommend alternative vision correction options.

ChatGPT-4.0

Yes, LASIK or RK surgery can cause keratoconus in certain individuals. Both procedures involve modifications to the cornea, which can lead to corneal ectasia, a condition that resembles keratoconus. While LASIK screening has improved over the years, many patients who underwent these surgeries in the past may still develop keratoconus as a complication 34. Additionally, the majority of ectasia cases occur after LASIK rather than PRK, indicating a higher risk associated with LASIK procedures 4.

The search results indicate that LASIK and RK surgeries can lead to corneal ectasia, a condition similar to keratoconus, due to the structural changes they cause in the cornea. This information is based on multiple sources that discuss the potential complications of these refractive surgeries.

Gemini

No, LASIK or RK surgery cannot cause keratoconus. These procedures are used to treat refractive errors like nearsightedness, farsightedness, and astigmatism by reshaping the cornea. They do not increase the risk of developing keratoconus.

However, if you have a **family history of keratoconus** or other risk factors, it's important to **discuss your options with your eye doctor** before considering LASIK or RK surgery. They can evaluate your individual situation and recommend the best treatment for you.<br><br>If you already have keratoconus, LASIK and RK surgery are **generally not recommended** as they may worsen the condition. There are other treatment options available for people with keratoconus, such as special contact lenses or corneal cross-linking.
**Copilot**
LASIK and RK (Radial Keratotomy) surgeries can potentially lead to a condition similar to keratoconus called **corneal ectasia**. This occurs when the cornea becomes too thin and weak after surgery, causing it to bulge outward.<br><br>Here are some key points:<br><br>• **LASIK**: This procedure reshapes the cornea by removing some of its tissue. If too much tissue is removed, it can weaken the cornea and lead to corneal ectasia.<br><br>• **RK Surgery**: This older procedure involves making radial cuts in the cornea to flatten it. Like LASIK, it can also weaken the cornea and potentially lead to ectasia[3].<br><br>Because of these risks, individuals with keratoconus or those at high risk for corneal thinning are generally not recommended to undergo LASIK or RK surgery
**Chatsonic**
LASIK (Laser-Assisted In Situ Keratomileusis) and RK (Radial Keratotomy) surgeries are not direct causes of keratoconus, but they can lead to a condition known as "post-surgical ectasia," which resembles keratoconus. Here's how these surgeries relate to keratoconus:<br><br>**LASIK and Keratoconus**<br><br>• **Corneal Thinning**: LASIK involves reshaping the cornea by removing tissue, which can weaken the corneal structure.<br><br>• **Risk of Ectasia**: In individuals with pre-existing corneal weakness or thin corneas, LASIK can increase the risk of developing ectasia, a condition similar to keratoconus.<br><br>**RK and Keratoconus**<br><br>• **Structural Changes**: RK involves making radial cuts in the cornea to flatten it, which can also weaken the corneal structure over time.<br><br>• **Progressive Changes**: Similar to LASIK, RK can lead to progressive corneal changes that mimic keratoconus.<br><br>**Risk Factors**

- **Pre-existing Conditions**: Individuals with undiagnosed keratoconus or other corneal abnormalities are at higher risk of developing ectasia after these surgeries.
- **Screening**: Thorough pre-surgical screening is crucial to identify individuals at risk.

Prevention and Management

- **Alternative Procedures**: Those with keratoconus or at risk of ectasia may be advised to consider alternative vision correction procedures.
- **Monitoring**: Regular follow-up is important to monitor corneal stability post-surgery.

In summary, while LASIK and RK do not cause keratoconus, they can lead to similar complications in susceptible individuals. Proper screening and monitoring are essential to minimize these risks.

Perplexity

No, LASIK or radial keratotomy (RK) surgery cannot directly cause keratoconus. However, there are a few important considerations:

Keratoconus Increases Risk of Post-LASIK Ectasia

While LASIK itself does not cause keratoconus, it can potentially unmask or exacerbate an underlying case of keratoconus that was previously undiagnosed. This is known as post-LASIK ectasia.Patients with undiagnosed, mild or subclinical keratoconus are at greatest risk for post-LASIK ectasia. The LASIK procedure may remove too much corneal tissue in these cases, leading to progressive corneal thinning and irregular astigmatism.

Screening for Keratoconus is Critical Before LASIK

To minimize the risk of post-LASIK ectasia, eye doctors perform thorough screening for keratoconus before approving a patient for LASIK. This includes:

- Detailed corneal topography mapping to detect any irregularities
- Measuring corneal thickness (pachymetry)
- Assessing family history and other risk factors

Patients with a confirmed diagnosis of keratoconus are typically not candidates for LASIK, as the procedure could exacerbate the condition.

Alternative Treatments for Keratoconus Patients

For keratoconus patients, treatments like corneal cross-linking, intacs corneal implants, or specialty contact lenses may be recommended instead of LASIK. In advanced cases, a corneal transplant may be needed to restore vision.In summary, while LASIK does not directly cause keratoconus, it is contraindicated in patients with the condition due to the risk of inducing further corneal instability and vision loss. Careful screening and alternative treatments are essential for keratoconus patients.

Question 9: Are There Multiple Forms of Keratoconus?

ChatGPT-3.5
Yes, there are multiple forms of keratoconus that vary in severity and progression:<br>1. **Forme Fruste Keratoconus**: This is a very mild, subclinical form of keratoconus where the changes in the cornea are subtle and may not cause noticeable symptoms. It can often be detected only through specialized imaging tests and rarely progresses.<br>2. **Nipple Cone**: This type of keratoconus features a small, steeply curved cone located centrally or slightly off-center on the cornea. It tends to progress rapidly and may cause significant visual distortion.<br>3. **Oval Cone**: The cone in this form is larger and more oval-shaped, often located below the center of the cornea. It leads to more pronounced astigmatism and vision problems.<br>4. **Globus Cone**: This is the most severe form of keratoconus, where a large portion of the cornea thins and bulges. It can affect almost half of the cornea, leading to significant vision impairment.<br>Each form can progress differently, with some staying mild and others advancing rapidly. Early detection and appropriate treatment can help manage the condition.
**ChatGPT-4.0**
Keratoconus has several forms, primarily categorized into four main types:<br>1. **Nipple Cone (Round Cone)**: This type affects a small area of the cornea but can have a steep slope, leading to significant visual deterioration .<br>2. **Oval Cone (Sagging Cone)**: This form impacts a larger area of the cornea and can cause complications such as rupturing of internal membranes and scarring, making contact lens fitting more difficult .<br>3. **Forme Fruste**: The mildest and most common type, often asymptomatic and detectable only through specialized imaging. It represents an early or non-progressive stage of keratoconus .<br>4. **Keratoglobus**: A rare form that affects the entire cornea, causing it to bulge outward and become thin and weak 4.<br>Other variations may exist, but these are the primary classifications recognized in clinical practice

Gemini
**Yes, there are different forms of keratoconus.** The condition can vary in severity and progression. Here are some of the main forms:<br><br>1. **Classic keratoconus:** This is the most common type, characterized by a gradual thinning and bulging of the central cornea.<br>2. **Penetrating keratoconus:** In this form, the cornea thins so much that it becomes very fragile and may even rupture.<br>3. **Infantile keratoconus:** This is a rare form that occurs in children and typically progresses more rapidly.<br>4. **Map-dot-fingerprint keratoconus:** This is a milder form with less severe corneal thinning.<br>5. **Pleomorphic keratoconus:** This form is characterized by irregular corneal changes, including thinning, bulging, and scarring.<br><br>The specific form of keratoconus can influence the severity of symptoms and the treatment options available. It's important to **work closely with your eye doctor** to determine the type of keratoconus you have and develop a personalized treatment plan.
**Copilot**
Yes, keratoconus can present in various forms and stages. It is a progressive eye condition that affects the cornea, causing it to thin and bulge into a cone-like shape. Here are the main stages and forms of keratoconus:<br><br>1. **Early Stage**: In the initial phase, the cornea starts to thin and bulge slightly. Vision may be slightly blurred or distorted, and frequent changes in eyeglass prescriptions might be needed.<br>2. **Moderate Stage**: As the condition progresses, the cornea becomes more irregular, leading to increased astigmatism and myopia (nearsightedness). Vision correction with glasses becomes less effective, and contact lenses might be required.<br>3. **Advanced Stage**: In severe cases, the cornea becomes significantly cone-shaped, causing substantial vision impairment. Scarring of the cornea can occur, further reducing vision quality. Specialized contact lenses or surgical interventions may be necessary.

There are also different classification systems used to stage keratoconus, such as the Amsler-Krumeich classification and the Belin ABCD classification, which take into account various measurements of the cornea.<br><br>If you have any specific concerns or symptoms, it's always best to consult with an eye care professional for a thorough evaluation and personalized advice. How are you managing your eye health currently?
**Chatsonic**
Yes, keratoconus can present in multiple forms, which vary in terms of severity, progression, and corneal shape. Here are the primary forms:<br><br>**1. Forme Fruste Keratoconus**<br><br>• **Mildest Form**: This is the earliest and mildest form of keratoconus. It may not cause significant vision problems and is often detected only through corneal topography.<br>• **Stable Condition**: It may remain stable and not progress to more severe forms.<br><br>**2. Nipple Cone**<br><br>• **Central Location**: Characterized by a small, steep cone located centrally or slightly off-center on the cornea.<br>• **Rapid Progression**: It can progress rapidly, leading to significant visual distortion.<br><br>**3. Oval Cone**<br><br>• **Larger Area**: The cone is larger and more oval-shaped, often located below the center of the cornea.<br>• **Moderate Impact**: This form can cause moderate to severe vision impairment.<br><br>**4. Keratoglobus**<br><br>• **Entire Cornea Affected**: Unlike the localized cones in other forms, keratoglobus involves thinning of the entire cornea.<br>• **Rare and Severe**: It is a rare and severe form of keratoconus, leading to significant vision problems.<br><br>**5. Pellucid Marginal Degeneration**<br><br>• **Peripheral Thinning**: Characterized by thinning of the cornea in a crescent shape near the lower periphery.<br>• **Unique Pattern**: It creates a "kissing doves" or "crab claw" pattern on corneal topography.

Each form of keratoconus requires specific management strategies, and early detection is crucial for effective treatment and vision preservation. Regular eye examinations and corneal imaging are key to identifying and monitoring these forms.
**Perplexity**
Yes, there are several different forms or types of keratoconus that are classified based on the shape and location of the corneal bulging:<br>Forme Fruste Keratoconus<br>• The mildest form where the disease is present but very early/mild or has already stopped progressing<br>• Can only be diagnosed through corneal topography mapping<br>• Usually only requires glasses or soft contact lenses for vision correction<br>Nipple Cone<br>• A small, isolated area of bulging at or near the center of the cornea<br>• Often causes substantial vision distortion since the bulging is in the pupil area<br>• Typically requires specialty small hard contact lenses, hybrids, or scleral lenses<br>Oval Cone<br>• The most common form with a larger area of bulging, usually inferiorly and temporally<br>• The distortion extends into the central cornea, decreasing vision with glasses or soft contacts<br>• Corrected with scleral or hybrid contact lenses<br>Globus Cone<br>• Extensive thinning and bulging affecting over 75% of the cornea<br>• The most severe but uncommon form<br>• More likely to require a corneal transplant (penetrating keratoplasty)<br>Other less common forms include keratoglobus (entire cornea thins and protrudes) and posterior keratoconus (posterior corneal surface steepens).The type of keratoconus, along with the severity, determines the appropriate treatment approach and vision correction options for each patient. Careful evaluation by an eye specialist is needed to determine the specific form present.
**Question 10: How Is Keratoconus Diagnosed?**

**ChatGPT-3.5**
Keratoconus is diagnosed through a series of eye exams and specialized tests. Common diagnostic methods include:<br><br>1. **Eye Exam**: A standard eye test can reveal early signs, such as irregular astigmatism or changes in vision that can't be corrected fully with glasses.<br>2. **Corneal Topography**: This is the most important and widely used test. It maps the shape of the cornea and can detect the irregular cone shape associated with keratoconus, often before symptoms are noticeable.<br>3. **Pachymetry**: This test measures the thickness of the cornea. Since keratoconus causes the cornea to thin, pachymetry helps in assessing the extent of the thinning.<br>4. **Slit-Lamp Examination**: A doctor uses a special microscope to examine the cornea's surface for any signs of thinning or scarring that could indicate keratoconus.<br>5. **Keratometry**: This measures the curvature of the cornea, helping to detect irregularities that could indicate keratoconus.<br><br>Early diagnosis is key to managing keratoconus effectively and preventing severe vision loss.
**ChatGPT-4.0**
The diagnostic methods for keratoconus include a comprehensive evaluation of medical history, a thorough eye examination, and specific tests to assess corneal shape and function. Key diagnostic tools are:<br><br>1. **Visual Acuity Tests**: These include the Snellen eye chart and phoropter assessments to determine vision clarity and prescription needs [cit:5].<br>2. **Slit-Lamp Examination**: This test uses a bright light and microscope to examine the cornea for signs of keratoconus, such as the presence of a Fleischer ring [cit:2][cit:5].<br>3. **Keratometry**: This measures the curvature of the cornea to identify astigmatism and irregularities [cit:5].<br>4. **Corneal Topography and Tomography**: These imaging techniques create detailed maps of the cornea's surface, helping to detect the characteristic changes associated with keratoconus [cit:4][cit:5].

5. **Pachymetry**: This measures the thickness of the cornea, which can be affected in keratoconus [cit:4].
6. **Genetic Testing**: Tools like AvaGen evaluate genetic markers associated with keratoconus, aiding in early detection [cit:3].

These methods collectively help in diagnosing keratoconus and determining its severity for appropriate management.

Gemini

Keratoconus is typically diagnosed through a comprehensive eye exam. Your eye doctor will likely perform the following tests:

1. **Visual acuity test:** This measures your ability to see clearly at different distances.
2. **Slit lamp exam:** This allows your eye doctor to examine the cornea and other structures of your eye.
3. **Corneal topography:** This test uses special imaging techniques to map the curvature of your cornea.
4. **Pachymetry:** This test measures the thickness of your cornea.
5. **Endothelial cell count:** This test measures the number of cells on the back surface of your cornea.

In some cases, additional tests may be necessary to confirm the diagnosis or rule out other conditions. These may include:

- **Corneal tomography:** This test provides a 3D image of your cornea.
- **Optical coherence tomography (OCT):** This test uses light waves to create detailed images of the cornea.

If you are experiencing symptoms of keratoconus, it's important to **see an eye doctor for a regular eye exam.** Early diagnosis can help prevent vision loss and allow for timely treatment.

Copilot

Diagnosing keratoconus involves several steps and specialized tests. Here are the common methods used:

1. **Medical and Family History**: Your eye doctor will start by reviewing your medical and family history to identify any risk factors or genetic predispositions.
2. **Eye Refraction**: This test measures how light bends as it passes through your eye. It helps determine the degree of vision correction needed and can reveal irregularities in the cornea.
3. **Slit-Lamp Examination**: A slit lamp is a microscope with a bright light used to examine the cornea, iris, lens, and the space between the iris and cornea. This test can detect abnormalities in the cornea's shape and structure.
4. **Keratometry**: This test measures the curvature of the cornea. It helps in detecting the cone-like bulge characteristic of keratoconus.
5. **Corneal Topography**: This is the most accurate method for diagnosing keratoconus. It creates a detailed map of the cornea's surface, highlighting any irregularities and the extent of the condition[12].
6. **Corneal Tomography**: This advanced imaging technique provides a three-dimensional view of the cornea, allowing for a more detailed assessment of its thickness and shape[2].

These tests help in diagnosing keratoconus early and monitoring its progression. If you suspect you have keratoconus or are experiencing symptoms like blurred vision or frequent changes in your eyeglass prescription, it's important to consult an eye care professional for a thorough evaluation.

Chatsonic

Diagnosing Keratoconus

Keratoconus is diagnosed through a combination of patient history, clinical examination, and specialized diagnostic tests. Here are the steps typically involved in diagnosing keratoconus:

Patient History

- **Symptoms**: Patients may report blurring of vision, distortion of images, increased light sensitivity, difficulty with night vision, or a need for frequent changes in eyeglass prescription.

Visual Acuity Test

- **Snellen Chart**: Standard eye charts are used to measure how well the patient can see at distances.

Slit-Lamp Examination

- **Corneal Appearance**: An eye doctor will use a slit lamp to examine the cornea for signs of keratoconus, such as thinning, cone-like shape, or Fleischer ring (an iron-colored ring around the cornea).

Corneal Topography

- **Mapping the Cornea**: This non-invasive imaging technique creates a detailed map of the curvature of the cornea. It can show patterns typical of keratoconus, such as steepening or thinning of the cornea.

Corneal Pachymetry

- **Corneal Thickness Measurement**: Pachymetry measures the thickness of the corneal tissue. Thinning of the cornea is a hallmark of keratoconus.

Corneal Tomography

- **3D Corneal Shape**: Corneal tomography provides a three-dimensional map of the cornea, which can help in diagnosing and assessing the progression of keratoconus.

Refraction

- **Measuring Prescription**: A detailed measurement of the eyes' refractive error can help in assessing the degree of astigmatism and myopia, which are often seen in keratoconus.

Other Diagnostic Tests

- **Biomicroscopy**: High-resolution imaging to view detailed corneal structure.
- **Specular or Confocal Microscopy**: To look at the cellular changes in the cornea.

Summary

Diagnosis of keratoconus is multifaceted, involving a detailed eye examination, patient history assessment, and advanced corneal imaging techniques. Early diagnosis is crucial, as it can halt or slow down the progression of the disease with appropriate treatment.

Perplexity

Keratoconus is diagnosed through a comprehensive evaluation that includes a combination of patient history, physical examination, and specialized diagnostic procedures. Here’s an overview of the diagnostic process:

Patient History

- **Visual Symptoms**: The clinician will gather information about the patient's visual symptoms, such as blurred or distorted vision, frequent changes in prescription, and any history of eye rubbing.

- **Family History**: A history of keratoconus in the family may also be noted, as there is a genetic component to the condition.

Physical Examination

- **Visual Acuity Tests**: Measurement of uncorrected and best-corrected visual acuity to assess the extent of vision impairment.
- **Slit-Lamp Examination**: This allows for the observation of the cornea for signs such as thinning, irregularities, and the presence of Fleischer rings or Vogt striae, which are indicative of keratoconus.

Diagnostic Procedures

1. **Corneal Topography**: This non-invasive imaging technique creates a detailed map of the cornea's surface, detecting irregularities and early signs of keratoconus. It is particularly effective in identifying subtle changes before other clinical signs appear.
2. **Corneal Tomography**: A more advanced imaging method that provides detailed information about corneal thickness and curvature, enhancing the detection of keratoconus.
3. **Pachymetry**: This test measures the thickness of the cornea, which is crucial for diagnosing keratoconus, as affected areas typically show thinning.
4. **Retinoscopy**: This technique assesses the refractive error and may show a scissoring reflex, which is common in keratoconus.
5. **Other Imaging Techniques**: Emerging technologies like optical coherence tomography (OCT) and Scheimpflug imaging are being utilized for more precise assessments of corneal structure and to detect early keratoconus.

Conclusion

The diagnosis of keratoconus relies on a thorough evaluation combining clinical findings and advanced imaging techniques. Early detection is crucial for effective management and treatment, as it can help preserve vision and prevent progression of the condition. Regular eye examinations are essential for individuals at risk or showing symptoms of keratoconus.

Question 11: How Do You Measure the Severity of Keratoconus?

ChatGPT-3.5

The severity of keratoconus is typically measured based on corneal curvature, thickness, and the extent of visual impairment. Here are key factors used to assess its severity:

1. **Corneal Curvature (K-Readings)**: The steepness of the cornea is measured using a keratometer or corneal topography.
   - **Mild**: Corneal curvature below 45 diopters.
   - **Moderate**: Curvature between 45–52 diopters.
   - **Severe**: Curvature above 52 diopters.
2. **Corneal Thickness (Pachymetry)**: Thinner corneas generally indicate more advanced keratoconus.
   - Normal corneal thickness is about 540-550 microns, and thinning below 400 microns is considered severe.
3. **Visual Acuity**: This assesses the impact on vision using standard eye charts. If visual distortion can't be corrected with glasses or soft contact lenses, the keratoconus may be moderate to severe.
4. **Corneal Scarring**: The presence of scarring indicates advanced keratoconus and often correlates with more severe visual impairment.
5. **Corneal Shape (Topography Maps)**: The irregularity and asymmetry of the cornea, as visualized on topography maps, provide a detailed look at the disease's progression.

The combination of these measurements helps determine whether the keratoconus is mild, moderate, or severe, guiding appropriate treatment options.

ChatGPT-4.0

To measure the severity of keratoconus, several methods and criteria are utilized:

1. **Amsler-Krumeich Scale**: This is the oldest grading system, which assesses corneal thickness, anterior keratometric measurements, and refractive error to classify the severity of keratoconus.
2. **ABCD Grading System**: A more recent classification that incorporates average anterior radius of curvature (A), posterior average radius of curvature (B), thinnest pachymetry measurement (C), and best spectacle-corrected distance visual acuity (D).
3. **D-value**: This is a multimetric combination index that includes keratometric, pachymetric, and elevation parameters, provided by the Belin/Ambrosio Enhanced Ectasia Software of the Pentacam System.
4. **Corneal Tomography and Topography**: These imaging techniques create detailed shape maps of the cornea and measure its thickness, which are essential for diagnosing and monitoring keratoconus.

5. **Clinical Signs**: Observations such as asymmetric refractive error, irregular astigmatism, and corneal thinning are also critical in assessing the severity of the condition.

These methods collectively help in diagnosing keratoconus and determining its severity for appropriate management.

Gemini

he severity of keratoconus is typically measured using a combination of tests and measurements. This includes:

1. **Corneal topography:** This test maps the curvature of your cornea, helping to identify the extent of the cone-shaped bulge.
2. **Pachymetry:** This test measures the thickness of your cornea, which can indicate how thin the cornea has become.
3. **Endothelial cell count:** This test measures the number of cells on the back surface of your cornea. A decrease in endothelial cell count may indicate advanced keratoconus.
4. **Visual acuity:** This test measures your ability to see clearly at different distances. A decrease in visual acuity can be a sign of worsening keratoconus.

Several grading systems are used to classify the severity of keratoconus, including the Amsler Grading System and the Belin-Ambrósio Grading System. These systems take into account factors such as the shape of the cornea, the thickness of the cornea, and the extent of visual impairment.

Copilot

The severity of keratoconus is typically measured using several classification systems and diagnostic tools. Here are some of the key methods:

Classification Systems

Amsler-Krumeich Classification:

Stage 1: Mild myopia and/or astigmatism, slight corneal steepening.

Stage 2: Moderate myopia and/or astigmatism, more pronounced corneal steepening, and thinning.

Stage 3: Severe myopia and/or astigmatism, significant corneal steepening, and thinning.

Stage 4: Advanced keratoconus with scarring and significant visual impairment.

Belin ABCD Classification:

This system uses advanced measurements including the steepness of the front and back of the cornea, corneal thickness, and visual acuity

Corneal Topography:

This imaging technique maps the surface curvature of the cornea, helping to detect and monitor the progression of keratoconus.

Pachymetry:

Measures the thickness of the cornea, which is crucial as keratoconus causes thinning.

Keratometry:

Measures the curvature of the cornea to assess the degree of steepening.

Ocular Coherence Tomography (OCT):

Provides detailed cross-sectional images of the cornea, allowing for precise measurement of corneal thickness and curvature.

These methods help ophthalmologists determine the stage of keratoconus and tailor treatment plans accordingly. If you have any more questions or need further details, feel free to ask!

Chatsonic

Measuring the Severity of Keratoconus

The severity of keratoconus is typically assessed by evaluating the cornea's shape, structure, and impact on vision. Various clinical methods and classification systems are used to quantify the degree of keratoconus. Here are some of the key parameters and methods:

Corneal Topography and Tomography

- **Curvature**: The degree of corneal steepening is a critical factor. The steeper the cornea, the more severe the keratoconus.
- **Asymmetry**: The more asymmetric the corneal shape between the two eyes, the more severe the condition may be considered.

Corneal Pachymetry

- **Corneal Thickness**: Thinner corneas are indicative of more advanced keratoconus. Severe cases can have corneal thickness below 400 micrometers.

Visual Acuity

- **Corrected and Uncorrected**: A significant reduction in both corrected and uncorrected visual acuity can indicate more severe keratoconus.

Amsler-Krumeich Classification

A widely used system that stages keratoconus based on corneal curvature, corneal thickness, refractive error, and scarring:

- **Stage I**: Mild keratoconus with corneal thickness of more than 500 micrometers and a curvature of less than 48 diopters.
- **Stage II**: Moderate keratoconus with corneal thickness between 400 and 500 micrometers and curvature between 48 and 53 diopters.
- **Stage III**: Advanced keratoconus with corneal thickness between 300 and 400 micrometers and curvature between 53 and 55 diopters.
- **Stage IV**: Severe keratoconus with corneal thickness less than 300 micrometers and curvature greater than 55 diopters.

Scarring

- **Presence of Scars**: The presence and extent of corneal scarring can also be indicative of the disease's severity.

Corneal Hydrops

- **Acute Events**: The occurrence of corneal hydrops, where a sudden influx of fluid into the cornea leads to swelling and scarring, is associated with severe keratoconus.

Contact Lens Tolerance

- **Fitting Difficulties**: Severe keratoconus can make it difficult to fit contact lenses comfortably, which can impact the patient's quality of life.

Patient-Reported Outcomes

- **Quality of Life**: Surveys and questionnaires can assess the impact of keratoconus on daily activities, which can be considered when evaluating severity.

Summary

The severity of keratoconus is measured by a combination of objective clinical findings and patient-reported experiences. Corneal imaging plays a crucial role in assessing the structural changes in the cornea, while visual acuity tests and patient quality of life assessments help understand the functional impact of the disease. Identifying the stage of keratoconus is important for determining the most appropriate management and treatment strategies.

Perplexity

The severity of keratoconus is measured using various grading systems that assess corneal shape, thickness, and other clinical indicators. Here are the primary methods used to evaluate the severity of keratoconus:

1. Keratoconus Severity Score (KSS)

- The KSS is a scoring system that ranges from 0 to 5, where 0 indicates suspected keratoconus and 5 indicates severe keratoconus.
- It incorporates clinical markers and two corneal topographic indices: average corneal power and root mean square (RMS) error for higher-order Zernike terms. Clinical signs such as Vogt striae, Fleischer rings, and corneal scarring are also considered.

2. Belin ABCD Grading System

- This system evaluates keratoconus based on four factors:
  - **A**: Anterior corneal radius
  - **B**: Curvature of the thinnest corneal location's 3.0 mm central zone
  - **C**: Slimmest corneal pachymetry (thickness)
  - **D**: Best corrected distance visual acuity
- Each factor contributes to a comprehensive assessment of the disease's severity.

3. Alio-Shabayek System

- Based on the Amsler–Krumeich classification, this system assesses corneal thinning, keratometry readings, corneal scars, and front corneal deviations to determine severity.

4. Corneal Topography and Tomography

- **Corneal Topography**: Measures the curvature of the cornea, identifying the steepest part of the cornea at the height of the cone.
  - **Mild Keratoconus**: Steepest corneal curvature ≤ 48.00D
  - **Moderate Keratoconus**: Steepest corneal curvature 48.00D to 53.00D
  - **Advanced Keratoconus**: Steepest corneal curvature ≥ 53.00D.
- **Corneal Thinning**: The lowest corneal thickness is also measured to assess severity:
  - **Mild Keratoconus**: Lowest thickness ≥ 500µm
  - **Moderate Keratoconus**: Lowest thickness 300µm – 500µm
  - **Advanced Keratoconus**: Lowest thickness ≤ 300µm.

5. RETICS Classification

- This framework incorporates corneal biomechanical parameters, including hysteresis and resistance factors, along with optical and visual function variables, to provide a comprehensive assessment of keratoconus severity.

These grading systems and diagnostic tools provide a structured approach to evaluating keratoconus severity, which is crucial for determining appropriate treatment options and monitoring disease progression. Regular assessments help ensure timely interventions to preserve vision and manage the condition effectively.

Question 12: How Can I Treat My Keratoconus?

ChatGPT-3.5

Keratoconus treatment depends on the severity of the condition and how much it affects your vision. Here are the main treatment options:

1. **Eyeglasses or Soft Contact Lenses**: In the early stages, glasses or soft contact lenses can correct mild vision problems caused by keratoconus.
2. **Rigid Gas Permeable (RGP) Contact Lenses**: As keratoconus progresses, RGP lenses may be needed to provide clearer vision. These lenses sit on top of the irregular cornea, providing a smoother surface for light to pass through.
3. **Hybrid Contact Lenses**: These lenses have a hard center with a soft outer ring, combining comfort with the clearer vision provided by RGP lenses.
4. **Scleral or Semi-Scleral Lenses**: These larger lenses rest on the sclera (white part of the eye) and vault over the cornea, offering comfort and improved vision in more advanced cases.
5. **Corneal Cross-Linking (CXL)**: This procedure strengthens the corneal tissue by applying riboflavin (vitamin B2) drops and exposing the eye to UV light. It helps halt the progression of keratoconus by stabilizing the cornea.
6. **Intacs**: These are small, semi-circular implants placed within the cornea to flatten and reshape it, improving vision in mild to moderate cases of keratoconus.
7. **Corneal Transplant (Keratoplasty)**: In severe cases where other treatments are ineffective, a corneal transplant may be required. This involves replacing the damaged cornea with healthy donor tissue.
8. **Custom Soft Lenses**: Some soft lenses designed specifically for keratoconus can correct more advanced stages than regular lenses.

Early detection and intervention are important for managing keratoconus and preserving vision.

ChatGPT-4.0

To treat keratoconus, I will explore corrective lenses options, evaluate the suitability of corneal cross-linking, and consider surgical options like Intacs or corneal transplant, each in separate steps. Let's start with exploring corrective lenses options.

Corrective Lenses Options for Keratoconus Treatment

1. **Glasses**: In the early stages of keratoconus, glasses may help correct vision, although they become less effective as the condition progresses.
2. **Rigid Gas Permeable (RGP) Lenses**: These lenses provide a smooth refractive surface, improving vision by compensating for the irregular shape of the cornea.
3. **Scleral Lenses**: Larger than RGP lenses, scleral lenses vault over the cornea and rest on the sclera, providing comfort and stability for more advanced keratoconus.
4. **Hybrid Lenses**: These lenses combine a hard center with a soft outer ring, offering the clarity of RGP lenses with the comfort of soft lenses.
5. **Custom Soft Contact Lenses**: Designed specifically for keratoconus, these lenses can provide improved comfort and vision correction.

Next, I will evaluate the suitability of corneal cross-linking for the user.

I will now evaluate the suitability of corneal cross-linking for keratoconus treatment, as it is a common procedure used to halt the progression of the condition by strengthening the corneal tissue. This will help determine if it is an appropriate option for managing keratoconus.

Corneal Cross-Linking for Keratoconus Treatment

Corneal cross-linking is a minimally invasive procedure designed to strengthen the corneal tissue and halt the progression of keratoconus. Here's how it works and its suitability:

1. **Procedure**:
   - The cornea is saturated with riboflavin (vitamin B2) eye drops.
   - Ultraviolet (UV) light is applied to the cornea, activating the riboflavin and creating new bonds between collagen fibers.
2. **Benefits**:
   - Stabilizes the cornea and prevents further thinning and bulging.
   - Can be combined with other treatments, such as contact lenses, for improved vision correction.

3. **Suitability**:

- Ideal for patients with progressive keratoconus, especially in the early to moderate stages.
- Not typically recommended for advanced keratoconus where significant scarring or thinning has occurred.

4. **Considerations**:

- Recovery time varies, and patients may experience temporary discomfort or vision changes.
- Regular follow-up is necessary to monitor the cornea's stability and response to treatment.

Next, I will consider surgical options like Intacs or corneal transplant for keratoconus treatment.

I will now consider surgical options like Intacs or corneal transplant for keratoconus treatment, as these are typically reserved for more advanced cases where other treatments are insufficient. This will help determine if surgical intervention is necessary for managing keratoconus.

Surgical Options for Keratoconus Treatment

1. **Intacs**:

- **Procedure**: Intacs are small, arc-shaped implants inserted into the cornea to flatten it and improve vision.
- **Benefits**: Can reduce corneal irregularities and improve contact lens fit.
- **Suitability**: Suitable for patients with mild to moderate keratoconus who are not candidates for corneal cross-linking or contact lenses.

2. **Corneal Transplant (Keratoplasty)**:

- **Procedure**: Involves replacing the damaged cornea with a healthy donor cornea.
- **Types**: Penetrating keratoplasty (full-thickness transplant) or deep anterior lamellar keratoplasty (partial-thickness transplant).
- **Benefits**: Can restore vision in advanced keratoconus cases with significant scarring or thinning.
- **Suitability**: Reserved for severe cases where other treatments have failed.

These surgical options provide additional avenues for treating keratoconus, particularly in advanced stages where other methods are insufficient. Regular consultation with an eye care professional is essential to determine the most appropriate treatment plan.

Gemini

Treating Keratoconus: A Guide

Keratoconus is a progressive eye condition that causes the cornea to thin and bulge outward. While there's no cure, various treatments can help manage symptoms and prevent further progression.

Here are some common treatment options:

Contact Lenses

Soft contact lenses: Often used in the early stages of keratoconus.

Rigid gas permeable (RGP) contact lenses: Designed to fit the irregular shape of the cornea and provide better vision.

Hybrid lenses: Combine the comfort of soft lenses with the clarity of RGP lenses.

Scleral lenses: Cover the entire cornea and sclera, offering a stable platform for vision correction.

Corneal Cross-Linking (CXL)

Strengthens the cornea: This procedure uses ultraviolet light and riboflavin (vitamin B2) to stiffen the cornea and slow down its progression.

Implantable Ring Segments (INTACS)

Reshapes the cornea: Small plastic rings are surgically implanted into the cornea to help correct its shape and improve vision.

Corneal Transplant

Replaces the cornea: In severe cases, a corneal transplant may be necessary to restore vision.

Other Treatments

Glasses: Can be used in the early stages of keratoconus, but may not provide sufficient correction as the condition progresses.

Topical medications: Eye drops can help manage symptoms, such as dry eyes or inflammation.

It's important to consult with an eye care professional for a personalized treatment plan. They can assess the severity of your keratoconus and recommend the most appropriate options.

Copilot

Treating keratoconus involves several approaches, depending on the severity of the condition. Here are some common treatment options:

Non-Surgical Treatments

1. **Eyeglasses or Soft Contact Lenses**:
   - Effective in the early stages to correct mild vision problems.
2. **Rigid Gas Permeable (RGP) Contact Lenses**:
   - Provide a more stable vision correction by creating a smooth refractive surface over the irregular cornea.
3. **Scleral and Hybrid Lenses**:
   - These lenses vault over the cornea and rest on the sclera (white part of the eye), providing comfort and better vision.

Surgical Treatments

1. **Corneal Cross-Linking (CXL)**:
   - A minimally invasive procedure that strengthens corneal tissue to halt the progression of keratoconus.
2. **Intacs**:
   - Small, arc-shaped inserts placed in the cornea to flatten it and improve vision.
3. **Topography-Guided Conductive Keratoplasty (CK)**:
   - Uses radiofrequency energy to reshape the cornea.
4. **Corneal Transplant (Keratoplasty)**:
   - In advanced cases, a partial or full corneal transplant may be necessary.

Advanced Treatments

1. **Custom Soft Contact Lenses**:
   - Designed specifically for keratoconus patients to provide better comfort and vision.
2. **Deep Anterior Lamellar Keratoplasty (DALK)**:
   - A type of corneal transplant that preserves the inner layers of the cornea.

It's important to consult with an eye care professional to determine the best treatment plan for your specific condition. If you have any more questions or need further details, feel free to ask!

Chatsonic

Treatment Options for Keratoconus

The treatment for keratoconus depends on the severity of the condition and how quickly it is progressing. Here are some common treatment options:

Eyeglasses or Soft Contact Lenses

- For mild keratoconus, glasses or soft contact lenses can help to correct the mild refractive errors.

Specialized Contact Lenses

As the condition progresses, glasses may no longer provide the best vision correction. Specialized contact lenses are often the preferred treatment:

- **Rigid Gas Permeable (RGP) Lenses**: These hard lenses can provide a smooth surface over the irregular cornea, improving vision.
- **Hybrid Lenses**: With a hard center and soft periphery, hybrid lenses can offer the vision correction of RGP lenses with comfort closer to that of soft lenses.
- **Scleral Lenses**: Large-diameter lenses that rest on the sclera and vault over the irregular cornea can be more comfortable and provide better vision for some patients.

Corneal Collagen Cross-Linking (CXL)

- This procedure is designed to halt the progression of keratoconus. It involves using ultraviolet light and a photosensitizer (riboflavin, vitamin B2) to strengthen the corneal fibers.

Intacs

- Corneal inserts or Intacs can be surgically placed in the cornea to flatten it, improve vision, and potentially defer the need for a corneal transplant.

Corneal Transplant

In advanced cases where vision correction is no longer effective and the cornea is significantly scarred:

- **Penetrating Keratoplasty (PK)**: This is a full-thickness corneal transplant.
- **Deep Anterior Lamellar Keratoplasty (DALK)**: This procedure replaces only the front layers of the cornea, leaving the back layer intact.

Other Surgical Options

- **Topography-Guided Conductive Keratoplasty**: This procedure can regularize the corneal surface in some cases.
- **Intrastromal Corneal Ring Segments (ICRS)**: This can improve the corneal shape and vision.

Management of Associated Conditions

- **Allergies**: Managing eye allergies to reduce eye rubbing is an important aspect of keratoconus treatment.
- **Dry Eyes**: Use of lubricating eye drops can alleviate discomfort due to dryness.

Lifestyle Adjustments

- Avoiding eye rubbing is critical to prevent further progression of the disease.

Monitoring

- Regular follow-up appointments with an eye care professional are important to monitor the progression of the disease and the effectiveness of treatments.

Summary

Keratoconus treatment is highly individualized and can range from corrective lenses to surgical interventions. The goal is to provide the best possible vision correction, halt the progression of the disease, and maintain the health of the cornea. Regular monitoring by an ophthalmologist or optometrist specialized in corneal diseases is essential for managing keratoconus over time.

Perplexity

Treating keratoconus involves a range of options tailored to the severity of the condition and the individual's specific needs. Here are the primary treatment approaches:

Non-Surgical Treatments

1. **Eyeglasses**:

- In the early stages of keratoconus, glasses may be sufficient to correct vision, particularly for mild cases.

2. **Contact Lenses**:

- As the condition progresses, many patients require specialized contact lenses. Options include:
  - **Rigid Gas-Permeable Lenses**: These lenses are commonly used as they provide better vision correction by creating a smooth refractive surface over the irregular cornea.
  - **Scleral Lenses**: These larger lenses vault over the cornea and rest on the sclera, providing comfort and improved vision for patients with more advanced keratoconus.
  - **Hybrid Lenses**: Combining a rigid center with a soft outer ring, these lenses can offer a balance of comfort and vision correction.

3. **Corneal Collagen Cross-Linking (CXL)**:

- This procedure strengthens the cornea by using riboflavin (vitamin B2) and ultraviolet light, aiming to halt the progression of keratoconus. It is particularly beneficial for patients with early to moderate keratoconus.

4. **Intacs**:

- These are small, curved implants inserted into the cornea to flatten it and improve vision. This option is typically considered for patients who are not candidates for a corneal transplant but need better vision correction.

Surgical Treatments

1. **Corneal Transplant**:

- In severe cases where other treatments fail, a corneal transplant may be necessary. This involves removing part or all of the affected cornea and replacing it with a donor cornea.

2. **Deep Anterior Lamellar Keratoplasty (DALK)**:

- A type of corneal transplant that replaces only the front layers of the cornea, preserving the patient's endothelium (the inner layer), which can reduce the risk of complications.

3. **Photorefractive Keratectomy (PRK)**:

- In some cases, PRK may be performed in conjunction with CXL to reshape the cornea and improve vision.

Conclusion

The choice of treatment for keratoconus depends on the severity of the condition and the patient's visual needs. Most patients will not require surgery, and many can achieve satisfactory vision with contact lenses or non-surgical interventions. Regular follow-ups with an eye care professional are essential to monitor the condition and adjust treatment as necessary.

Question 13: What is the Best Treatment for Keratoconus?

ChatGPT-3.5

he best treatment for keratoconus depends on the severity of the condition, how much it affects your vision, and how fast it progresses. Here are the most effective treatments tailored to different stages of keratoconus:

1. Mild Keratoconus

- **Eyeglasses or Soft Contact Lenses**: Effective in early stages to correct mild vision distortion.

2. Moderate Keratoconus

- **Rigid Gas Permeable (RGP) Lenses**: Provide clearer vision by reshaping the cornea's irregular surface.
- **Hybrid or Scleral Lenses**: Offer improved comfort and stability compared to RGP lenses, especially for more advanced corneal irregularity.

3. Progressive or Moderate-to-Severe Keratoconus

- **Corneal Cross-Linking (CXL)**: The best treatment to stop the progression of keratoconus. It strengthens the cornea to prevent further thinning and bulging. Early use of this procedure is often recommended to prevent worsening of the condition.
- **Intacs**: Useful for moderate keratoconus to flatten the cornea and improve vision, often in combination with other treatments.

4. Severe or Advanced Keratoconus

- **Scleral or Semi-Scleral Lenses**: These large lenses vault over the cornea and rest on the sclera, offering comfort and clear vision even in advanced cases.
- **Corneal Transplant (Keratoplasty)**: In severe cases where scarring or extreme thinning occurs and other treatments are ineffective, a corneal transplant may be necessary.

Optimal Treatment:

For most patients with progressive keratoconus, **corneal cross-linking (CXL)** is considered the best option to halt disease progression. It can be combined with specialty lenses (RGP, scleral, or hybrid) to improve vision and quality of life.

Choosing the right treatment depends on your individual condition, and consultation with an eye specialist is crucial for determining the best approach.

ChatGPT-4.0

To determine the best treatment for keratoconus, I will first evaluate the severity of the condition using clinical markers and diagnostic tests, then consider individual patient factors such as age, lifestyle, and progression, and finally recommend the most suitable treatment option based on these evaluations. Let's proceed with evaluating the severity of keratoconus.

Step 1: Evaluate the Severity of Keratoconus

To evaluate the severity of keratoconus, clinical markers and diagnostic tests such as corneal topography, pachymetry, and keratometry are used. These tests assess the cornea's shape,

thickness, and curvature, providing a comprehensive understanding of the condition's progression.

- **Corneal Topography**: Creates a detailed map of the cornea's surface, highlighting irregularities and asymmetries.
- **Pachymetry**: Measures corneal thickness, which is crucial for determining the extent of thinning.
- **Keratometry**: Assesses the curvature of the cornea, identifying steepening that indicates keratoconus.

These diagnostic tools help classify the severity of keratoconus, ranging from mild to advanced stages, guiding the treatment approach.

Step 2: Consider Individual Patient Factors

Next, I will consider individual patient factors such as age, lifestyle, and progression of keratoconus to tailor the treatment recommendation.

To determine the best treatment for keratoconus, it's essential to evaluate the severity of the condition and consider individual patient factors, as these will guide the choice of treatment, ensuring it is tailored to the patient's specific needs and circumstances.

To determine the best treatment for keratoconus, it's crucial to evaluate the severity of the condition and consider individual patient factors, as these will guide the choice of treatment, ensuring it is tailored to the patient's specific needs and circumstances.

Gemini

The best treatment for keratoconus depends on the severity of the condition. There is no one-size-fits-all approach. Your eye doctor will carefully evaluate your individual situation and recommend the most appropriate treatment.

Common treatment options include:

- **Contact lenses:** Special contact lenses, such as rigid gas permeable (RGP) lenses or hybrid lenses, can help correct the irregular shape of the cornea and improve vision.
- **Corneal cross-linking (CXL):** This procedure uses ultraviolet light to strengthen the cornea and slow down its progression. It is often used in combination with RGP lenses.
- **Intacs:** These are tiny plastic rings that are implanted into the cornea to help reshape it and improve vision.
- **Corneal transplantation:** In severe cases, a corneal transplant may be necessary to replace the damaged cornea with a healthy donor cornea.

The choice of treatment will depend on factors such as:

- **The severity of keratoconus:** More advanced cases may require more invasive treatments.
- **Your overall eye health:** Other eye conditions or systemic diseases may influence the treatment options.
- **Your personal preferences:** You may have a preference for certain treatments based on comfort, lifestyle, or other factors.

It's important to work closely with your eye doctor to determine the best treatment for you and to follow their instructions carefully. Regular follow-up appointments are also essential to monitor the progression of your keratoconus and ensure that your treatment is effective.

Copilot

The best treatment for keratoconus depends on the severity of the condition and how much it affects your vision. Here are some common treatments:

1. Eyeglasses or Soft Contact Lenses: In the early stages, vision problems can often be corrected with glasses or soft contact lenses[1].
2. Rigid Gas Permeable (RGP) Contact Lenses: As keratoconus progresses, RGP lenses may be needed to provide clearer vision[1].
3. Scleral Lenses: These larger lenses sit on the sclera (the white part of the eye) and vault over the cornea, providing comfort and improved vision[1].
4. Corneal Cross-Linking (CXL): This procedure strengthens the corneal tissue to halt the progression of keratoconus. It involves applying riboflavin (vitamin B2) to the cornea and then activating it with UV light[2].
5. Implantable Ring Segments (Intacs): These small, curved devices are inserted into the cornea to help flatten it and improve vision[3].
6. Corneal Transplant: In advanced cases, a corneal transplant may be necessary to restore vision[1].

It’s important to consult with an eye care professional to determine the most appropriate treatment for your specific condition. Have you been diagnosed with keratoconus, or are you seeking information for someone else?

Chatsonic

The "best" keratoconus treatment varies for each individual and depends on the specific characteristics and progression of the condition. There is no one-size-fits-all solution, and treatment plans are usually tailored to each patient's unique circumstances. Some considerations for determining the most suitable treatment include:

Severity of Keratoconus

- The stage and severity of keratoconus, as determined through corneal topography, pachymetry, and visual acuity assessments.

Progression Rate

- The rate at which the disease is progressing, as rapid progression may necessitate more aggressive treatment.

Patient's Age and Lifestyle

- The patient's age, visual demands, and lifestyle factors can influence treatment decisions.

Patient's Comfort and Preference

- The patient's comfort with various treatment options, such as contact lenses, and their preference for non-invasive or surgical interventions.

Financial Considerations

- The availability of insurance coverage or financial resources for treatments such as corneal cross-linking or surgical procedures.

Collaborative Decision-Making

- Effective communication and shared decision-making between the patient and their eye care provider to arrive at a treatment plan that aligns with the patient's goals and expectations.

Possible Treatment Options

- **Corneal Cross-Linking (CXL)**: Often considered the standard of care for halting the progression of keratoconus.
- **Specialized Contact Lenses**: Rigid gas permeable (RGP), hybrid, or scleral lenses for vision correction.
- **Intacs**: Surgical implants to reshape the cornea and improve vision.
- **Corneal Transplant**: In advanced cases, a full-thickness or partial corneal transplant may be necessary.
- **Topography-Guided Conductive Keratoplasty**: An option for some patients to regularize the corneal surface.

Conclusion

The best keratoconus treatment is ultimately determined through a comprehensive assessment of the individual's condition and needs, in consultation with an experienced eye care professional. The chosen treatment should aim to provide optimal vision correction, halt the progression of the disease, and ensure the long-term health of the cornea. Regular follow-up and monitoring are essential to evaluate the effectiveness of the chosen treatment and make any necessary adjustments over time.

Perplexity

The best treatment for keratoconus depends on the severity of the condition and the individual patient's needs. However, scleral contact lenses are currently considered the most effective treatment option for most patients:

Scleral Contact Lenses

- Scleral lenses are large diameter gas permeable contact lenses that vault over the irregular cornea without touching it.
- They provide excellent vision correction and comfort by resting on the white part of the eye (sclera) rather than the sensitive cornea.
- Scleral lenses can be highly customized to achieve optimal vision and fit for each individual patient.
- They are the most popular and successful treatment option among keratoconus specialists.

Other Treatment Options

- In early stages, glasses or soft contact lenses may provide adequate vision correction.
- Corneal collagen cross-linking (CXL) is used to halt the progression of keratoconus, especially in early to moderate cases.
- Intacs corneal implants can help flatten the cornea and improve vision in some patients.
- In advanced cases with severe corneal scarring or thinning, a corneal transplant may be necessary to restore vision.

The choice of treatment depends on the specific type and severity of keratoconus, as well as the patient's visual needs and preferences. Regular follow-up with an eye care professional is crucial to monitor the condition and adjust treatment as needed. With proper management, most patients with keratoconus can maintain good vision and quality of life.

Question 14: How Can I Stop My Keratoconus From Getting Worse?

ChatGPT-3.5

To stop keratoconus from getting worse, early detection and intervention are key. Here are the most effective strategies:

1. Corneal Cross-Linking (CXL)

- **Best option for halting progression**: Corneal cross-linking (CXL) is a minimally invasive procedure that strengthens the cornea by using riboflavin (vitamin B2) and UV light. This treatment is designed to stop keratoconus from worsening by preventing further thinning and bulging of the cornea. Early treatment with CXL can prevent severe vision deterioration.

2. Avoid Eye Rubbing

- **Reduce physical strain on the cornea**: Frequent or vigorous eye rubbing is linked to worsening keratoconus. If you have allergies or conditions that cause itchy eyes, managing them with antihistamines or lubricating eye drops can reduce the urge to rub your eyes.

3. Regular Monitoring

- **Frequent checkups with your eye doctor**: Regular eye exams, including corneal topography and pachymetry, can help track changes in the shape and thickness of your cornea. Early detection of worsening keratoconus allows timely intervention.

4. Use Specialty Lenses

- **Properly fitted contact lenses**: Rigid gas permeable (RGP), hybrid, or scleral lenses can help manage vision problems and support the cornea by providing a smoother surface. Proper lens fitting by an eye specialist is essential to avoid irritation or damage to the cornea.

5. Protect Your Eyes

- **Wear UV-protective sunglasses**: Protecting your eyes from excessive UV light can prevent further damage. Also, avoid activities that could cause trauma to your eyes, like certain contact sports, without appropriate protective gear.

By following these steps and working closely with your eye specialist, you can significantly reduce the risk of keratoconus progression and preserve your vision.

ChatGPT-4.0
Based on the severity of keratoconus and individual patient factors, the most suitable treatment option can be recommended. This involves considering the stage of keratoconus, the patient's age, lifestyle, and how rapidly the condition is progressing. Let's proceed with recommending the treatment. To stop keratoconus from getting worse, the most suitable treatment option based on severity and patient factors includes:<br>1. Corneal Cross-Linking: Ideal for early to moderate keratoconus, this procedure strengthens the cornea and halts progression.<br>2. Specialized Contact Lenses: Rigid gas permeable or scleral lenses can provide better vision correction and comfort.<br>3. Regular Monitoring: Frequent eye exams to monitor changes and adjust treatment as needed.<br>4. Avoid Eye Rubbing: Reducing eye rubbing can prevent further damage to the cornea.<br>5. Protective Eyewear: Using sunglasses to protect against UV light may help reduce progression.<br>These strategies, tailored to the individual's condition and lifestyle, can effectively manage keratoconus and prevent worsening.
**Gemini**
**While there is no cure for keratoconus, there are steps you can take to help slow its progression and prevent it from getting worse:**<br>1. **Regular eye exams:** See your eye doctor regularly to monitor the progression of your keratoconus and receive timely treatment.<br>2. **Wear appropriate contact lenses:** If prescribed, wear the recommended contact lenses consistently and follow your eye doctor's instructions.<br>3. **Avoid eye rubbing:** Excessive eye rubbing can weaken the cornea and contribute to the progression of keratoconus.<br>4. **Protect your eyes from UV rays:** Wear sunglasses with UV protection to shield your eyes from harmful sunlight.<br>5. **Manage allergies:** If you have allergies, take steps to manage them to reduce eye irritation and inflammation.

6. **Consider corneal cross-linking (CXL):** This procedure can help strengthen the cornea and slow down the progression of keratoconus.

7. **Limit screen time:** Excessive screen time can lead to eye strain and fatigue, which may contribute to the progression of keratoconus.

8. **Maintain a healthy lifestyle:** A balanced diet, regular exercise, and adequate sleep can help support your overall health and eye health.

By following these steps, you can help reduce the risk of keratoconus worsening and improve your overall eye health.

Copilot

Managing keratoconus effectively involves a combination of medical treatments and lifestyle adjustments. Here are some key strategies:

1. **Avoid Eye Rubbing: This is crucial as rubbing your eyes can worsen keratoconus[12].**

2. **Corneal Cross-Linking (CXL): This medical procedure can help stabilize the cornea and prevent further progression[3].**

3. **Specialized Contact Lenses: Scleral or rigid gas permeable lenses can help improve vision and manage keratoconus[1].**

4. **Protect Your Eyes: Wear 100% UV-blocking sunglasses to protect your eyes from harmful UV rays[2].**

5. **Healthy Diet: Consuming foods rich in antioxidants, like leafy greens and berries, can support overall eye health[2].**

6. **Regular Eye Exams: Frequent check-ups with your eye doctor are essential to monitor the condition and adjust treatments as needed[1].**

If you're unsure about any advice or treatment, consider seeking a second opinion from a keratoconus specialist. How are you currently managing your condition?

Chatsonic

To stop the progression of keratoconus and prevent it from getting worse, several approaches can be considered. These measures are aimed at stabilizing the cornea and preserving vision. Here are some strategies to help halt the progression of keratoconus:

Corneal Cross-Linking (CXL)

- **Strengthening the Cornea**: CXL is a minimally invasive procedure that uses ultraviolet light and riboflavin (vitamin B2) to strengthen the corneal collagen, potentially preventing further thinning and bulging.

Regular Monitoring

- **Ongoing Evaluation**: Regular visits to an eye care professional for comprehensive eye examinations and corneal imaging to monitor changes in the cornea's shape and thickness.

Avoiding Eye Rubbing

- **Minimizing Irritation**: Rubbing the eyes can exacerbate the progression of keratoconus. Strategies to minimize eye rubbing, such as addressing allergies or dry eye symptoms, can be beneficial.

Contact Lens Management

- **Proper Fit and Usage**: Ensuring that contact lenses, especially rigid gas permeable (RGP), hybrid, or scleral lenses, are fitted properly and used as advised by the eye care professional.

UV Protection

- **Sunglasses**: Wearing sunglasses that provide ultraviolet (UV) protection to shield the eyes from harmful UV rays, which can potentially contribute to corneal thinning.

Managing Allergies and Dry Eyes

- **Minimizing Irritants**: Addressing underlying conditions such as eye allergies and dry eyes, as these can lead to increased eye rubbing and irritation.

Lifestyle Modifications

- **Healthy Habits**: Emphasizing good eye care practices, such as maintaining a healthy diet and avoiding habits that can strain the eyes, such as excessive screen time.

Summary

Implementing these measures, in consultation with an eye care professional, can contribute to stabilizing keratoconus and preventing its progression. Corneal cross-linking, in particular, has been shown to be effective in halting the advancement of the disease. Regular monitoring and proactive management of associated conditions can help preserve vision and maintain the health of the cornea over time.

Perplexity

To stop keratoconus from worsening, several strategies can be employed, focusing on early intervention and management. Here are the key approaches:

1. Regular Eye Examinations

- **Frequent Monitoring**: Regular visits to an eye care professional are essential to monitor the condition and detect any changes in vision or corneal shape. Early detection can lead to timely interventions that may prevent progression.

2. Corneal Collagen Cross-Linking (CXL)

- **CXL Procedure**: This minimally invasive treatment strengthens the cornea by using riboflavin (vitamin B2) and ultraviolet light to create new cross-links between collagen fibers. This process helps stabilize the cornea and can significantly slow or halt the progression of keratoconus, especially in early to moderate cases.
- **Post-Operative Care**: Following the procedure, it's crucial to adhere to your ophthalmologist's postoperative instructions, which may include using antibiotic and anti-inflammatory eye drops, avoiding eye rubbing, and attending follow-up appointments to monitor healing.

3. Avoid Eye Rubbing

- **Minimize Trauma**: Chronic eye rubbing can exacerbate keratoconus. It's important to avoid this habit, which is often linked to allergies and irritation. Managing allergies effectively can reduce the urge to rub the eyes.

4. Specialty Contact Lenses

- **Contact Lens Options**: As keratoconus progresses, specially designed contact lenses, such as rigid gas-permeable lenses or scleral lenses, can help improve vision and comfort. These lenses create a smoother optical surface over the irregular cornea.

5. Lifestyle Modifications

- **Protective Eyewear**: Wearing sunglasses to protect against UV exposure and reducing irritants can help maintain corneal health.
- **Healthy Habits**: Maintaining overall health, including a balanced diet and hydration, supports eye health and may help manage keratoconus.

6. Emerging Treatments

- **Investigational Options**: Research is ongoing into other potential treatments, such as copper sulfate eye drops and extracellular vesicles, which may offer non-invasive options for preventing keratoconus progression in the future.

By following these strategies and working closely with an eye care professional, individuals with keratoconus can effectively manage their condition and reduce the risk of progression. Early detection and intervention are crucial for achieving the best outcomes.

Question 15: Is Keratoconus Always Progressive?

ChatGPT-3.5

Keratoconus is **usually progressive**, but not always. The rate and extent of progression can vary significantly from person to person. Here are some key points about its progression:

1. **Typically Progressive**: In most cases, keratoconus starts in the teenage years or early adulthood and worsens over time. It tends to progress for 10 to 20 years before stabilizing. During this time, the cornea thins and bulges more, leading to increased vision distortion.
2. **Variable Progression**: While many people experience steady worsening, others may have a slow or minimal progression. Some individuals may see their keratoconus stabilize naturally, especially later in life.
3. **Mild or Forme Fruste Keratoconus**: In cases of mild or subclinical keratoconus (forme fruste), the condition may not progress significantly and could remain stable for years without requiring aggressive treatment.
4. **Influencing Factors**: Progression can be influenced by factors such as genetics, environmental factors (like frequent eye rubbing), and underlying medical conditions.

Early intervention with treatments like **corneal cross-linking (CXL)** can help halt progression in most cases, even if keratoconus is progressive by nature.

ChatGPT-4.0

Keratoconus is generally considered a progressive eye condition, meaning it can worsen over time, particularly during adolescence and early adulthood. However, it does not always progress indefinitely. Some patients may experience stabilization or even improvement in their condition, especially with treatments like corneal cross-linking (CXL), which has been shown to halt progression in a significant number of cases . While continued progression is possible, it is not common, and studies indicate that the rate of progression after CXL can vary widely 4. Regular eye exams are essential for monitoring the condition and determining the need for treatment .

Based on the search results, keratoconus is generally progressive, but treatments like corneal cross-linking can halt its progression, and some patients may experience stabilization. Regular monitoring is essential to manage the condition effectively.

**Gemini**
**No, keratoconus is not always progressive.** While it is a degenerative condition that typically worsens over time, the rate of progression can vary significantly from person to person.<br><br>**In some cases, keratoconus may stabilize or even stop progressing.** This is especially true when early intervention and appropriate treatment are received. For example, corneal cross-linking (CXL) can help strengthen the cornea and slow down the progression of the condition.<br><br>**However, it's important to note that keratoconus can still progress even after treatment.** Regular eye exams and follow-up care are essential to monitor the condition and ensure that your treatment is effective.
**Copilot**
Keratoconus is often progressive, but the rate and extent of progression can vary widely among individuals. For some, the condition may stabilize after a certain point, while for others, it may continue to worsen over time. Factors influencing progression include:<br><br>1. **Age**: Younger individuals tend to experience faster progression.<br>2. **Genetics**: Family history can play a role in how the condition develops.<br>3. **Eye Rubbing**: Frequent eye rubbing can exacerbate the condition.<br><br>Regular monitoring and early intervention are key to managing keratoconus effectively. Have you noticed any changes in your vision recently?
**Chatsonic**
Keratoconus is typically considered a progressive condition, meaning that it tends to worsen over time. However, the rate of progression can vary significantly among individuals. While some people may experience a gradual advancement of the condition, others may have a more stable course with minimal changes over long periods. Factors that can influence the progression of keratoconus include:

Age of Onset

- Keratoconus often begins during the teenage years or early twenties. The rate of progression may be more pronounced during this period.

Rate of Progression

- The speed at which keratoconus advances can differ from person to person. Some individuals may experience rapid changes in corneal shape and vision, while others may have a more gradual progression.

Environmental and Behavioral Factors

- Eye rubbing, poorly fitted contact lenses, and certain environmental factors may contribute to the acceleration of keratoconus.

Treatment and Management

- Early intervention with treatments such as corneal cross-linking and appropriate management strategies can help slow or halt the progression of keratoconus.

Genetic and Biological Factors

- Genetic predisposition and individual variations in corneal structure and biomechanics can influence the rate of progression.

Conclusion

While keratoconus is typically characterized by progression, the extent and speed of advancement can differ widely. Early diagnosis, regular monitoring, and proactive management are essential in addressing the condition and mitigating its progression. Interventions such as corneal cross-linking have shown promise in halting the advancement of keratoconus, emphasizing the importance of timely and appropriate treatment.

Perplexity

Keratoconus is generally considered a progressive condition, but it does not always continue to worsen indefinitely. Here are key points regarding the progression of keratoconus:

Typical Progression

- **Onset and Development**: Keratoconus often begins in the teenage years or early adulthood and can progress during the 20s and 30s. The condition is characterized by the thinning and bulging of the cornea, leading to visual impairment.

- **Stabilization**: For many individuals, keratoconus stabilizes after reaching around 25 years of age. Studies indicate that the majority of patients experience a cessation of progression after this age, particularly if they have received appropriate treatment and management.

Factors Influencing Progression

- **Eye Rubbing**: Chronic eye rubbing is a significant risk factor that can exacerbate keratoconus. Patients are advised to minimize this behavior, especially if it is linked to allergies or irritation.
- **Contact Lens Fit**: Poorly fitted contact lenses can contribute to the progression of keratoconus. Proper fitting and regular follow-ups with an eye care professional are essential for managing the condition effectively.
- **Pregnancy**: In some cases, hormonal changes during pregnancy may lead to progression, but this is not universally experienced.

Treatment Options

- **Corneal Collagen Cross-Linking (CXL)**: This procedure has been shown to halt or significantly slow the progression of keratoconus by strengthening the corneal structure. It is particularly beneficial for patients diagnosed in the early to moderate stages of the disease.
- **Regular Monitoring**: Routine eye examinations are crucial for tracking the condition and making timely adjustments to treatment plans. Early interventions can help maintain visual acuity and prevent further deterioration.

In summary, while keratoconus is typically progressive, many patients experience stabilization after a certain age, especially with appropriate management and treatment. Regular monitoring and interventions like corneal cross-linking can significantly impact the progression of the disease.

Question 16: Does Keratoconus Cause Eye Pain?

ChatGPT-3.5

Keratoconus itself typically **does not directly cause eye pain**, but discomfort or irritation can occur due to several factors related to the condition:

1. **Contact Lens Discomfort**: As keratoconus progresses, the cornea becomes more irregular, making it difficult to fit contact lenses properly. Poorly fitting lenses, especially rigid gas permeable (RGP) or scleral lenses, can cause eye discomfort, dryness, or irritation.
2. **Eye Strain**: The distortion of vision may cause eye strain, headaches, or fatigue as you strain to see clearly.

3. **Advanced Keratoconus (Hydrops)**: In very advanced cases, a condition called **corneal hydrops** can occur. This happens when fluid from inside the eye leaks into the cornea due to a tear in the Descemet membrane, causing sudden swelling, blurred vision, and sometimes significant pain.

4. **Eye Rubbing**: People with keratoconus often rub their eyes because of irritation or itchiness, which can lead to discomfort or even damage to the cornea, worsening the condition.

While mild discomfort is common, severe pain is not typical unless complications arise.

ChatGPT-4.0

Keratoconus does not always cause eye pain; however, its symptoms can lead to discomfort. The most common symptoms include blurred vision, astigmatism, and corneal thinning, which may result in sensitivity to light and glare. Some individuals may experience eye irritation or headaches associated with their vision problems, but severe eye pain is not a typical symptom of keratoconus itself . If a person with keratoconus experiences sudden eye pain, it is advisable to consult a specialist immediately .

The search results indicate that while keratoconus primarily causes vision-related symptoms, it can lead to discomfort such as eye irritation or headaches, but not typically severe eye pain. If severe pain occurs, it may indicate a complication, necessitating immediate medical attention. This understanding is based on the gathered information from reliable medical sources.

Gemini

Keratoconus typically does not cause significant eye pain. However, some patients may experience mild discomfort, especially during flare-ups or when the condition is severe.

If you are experiencing eye pain or discomfort along with other symptoms of keratoconus, it's important to **see an eye doctor** to rule out other potential causes and determine the best course of treatment.

Common causes of eye pain in people with keratoconus can include:

- **Dry eye syndrome:** This condition can occur when the eyes don't produce enough tears or when the tears evaporate too quickly.
- **Corneal scarring:** As keratoconus progresses, the cornea may become scarred, which can lead to discomfort.

- **Eye infections:** People with keratoconus may be more susceptible to eye infections, which can cause pain and redness.

If you are experiencing eye pain or discomfort, it's important to seek medical attention promptly.

Copilot

Keratoconus typically does not cause eye pain directly. However, some individuals may experience discomfort or irritation due to:

1. **Contact Lens Wear**: Ill-fitting lenses can cause discomfort.
2. **Eye Strain**: Trying to focus with distorted vision can lead to eye strain and headaches.
3. **Dry Eyes**: Keratoconus can sometimes be associated with dry eyes, which can cause irritation.

If you're experiencing significant pain, it's important to consult with your eye doctor to rule out other potential issues. Have you been feeling any discomfort lately?

Chatsonic

Keratoconus itself typically does not directly cause eye pain. However, individuals with keratoconus may experience discomfort or pain due to associated conditions or complications. Here are some factors to consider:

Corneal Abrasions and Scarring

- The irregular shape of the cornea in keratoconus can make it more susceptible to abrasions, which can cause pain and discomfort. Additionally, corneal scarring, which can occur in advanced cases, may lead to discomfort.

Dry Eye Syndrome

- Many individuals with keratoconus also experience dry eye symptoms, which can include sensations of grittiness, burning, and discomfort.

Contact Lens Discomfort

- Improperly fitted contact lenses, which are commonly used to correct vision in keratoconus, can cause irritation and discomfort.

Eye Rubbing

- The urge to rub the eyes, which is common in keratoconus due to associated itching or discomfort, can lead to further irritation and pain.

Other Conditions

- Individuals with keratoconus may also have other ocular conditions that can cause eye pain, such as allergies, conjunctivitis, or glaucoma.

Conclusion

While keratoconus itself may not be a direct cause of eye pain, associated conditions, complications, and habits such as eye rubbing can contribute to discomfort. It's important for individuals with keratoconus to discuss any eye pain or discomfort with their eye care professional to identify the underlying causes and determine appropriate management strategies.

Perplexity

Keratoconus does not typically cause eye pain directly, but it can lead to discomfort and other symptoms that may be perceived as pain. Here are some key points regarding the relationship between keratoconus and eye discomfort:

Symptoms Associated with Keratoconus

- **Visual Distortions**: Patients often experience blurred or distorted vision, which can lead to eye strain and discomfort, especially when trying to focus on objects.
- **Light Sensitivity**: Increased sensitivity to bright lights and glare can cause discomfort, particularly in bright environments or at night.
- **Eye Irritation**: Some individuals may experience irritation or a feeling of pressure in the eyes, which can be uncomfortable.
- **Headaches**: The visual strain from trying to compensate for distorted vision can lead to headaches, which may be interpreted as eye pain.
- **Sudden Worsening of Vision**: In some cases, keratoconus can lead to sudden changes in vision quality, which might cause distress and discomfort.

Conclusion

While keratoconus itself does not usually cause direct eye pain, the associated symptoms can lead to discomfort and a sensation of strain. If you experience significant discomfort or pain, it is important to consult an eye care professional for a thorough evaluation and appropriate management options. Regular monitoring and treatment can help alleviate symptoms and improve quality of life for individuals with keratoconus.

Question 17: Can Keratoconus Go Away On Its Own?
**ChatGPT-3.5**
No, keratoconus does not go away on its own. It is a progressive condition, meaning the cornea continues to thin and bulge over time. However, the rate of progression varies, and in some cases, it may stabilize naturally after several years. This usually happens later in life when the condition slows down or stops worsening, but it does not reverse or heal without intervention.<br><br>While it can't go away, treatments like **corneal cross-linking (CXL)** can halt the progression and stabilize the cornea, preventing further deterioration. Vision correction with specialty contact lenses can manage symptoms, but the underlying condition remains.
**ChatGPT-4.0**
No, keratoconus does not resolve on its own. It is a chronic eye disorder that typically progresses over time and does not fade without intervention. The condition is characterized by a progressive thinning and bulging of the cornea, and while treatments can help manage the symptoms and slow progression, there is no natural or medical way to reverse it 24.<br><br>The search results clearly indicate that keratoconus does not resolve on its own and is a chronic condition that typically requires intervention for management. There is no natural or medical way to reverse it, and treatments are necessary to slow its progression and manage symptoms. I will now summarize this information concisely.
**Gemini**
**No, keratoconus does not typically go away on its own.** It is a progressive condition that tends to worsen over time. While there are treatment options available to help manage the condition and prevent vision loss, there is no cure for keratoconus.<br><br>However, the rate of progression can vary significantly from person to person, and in some cases, it may stabilize or even slow down. Early diagnosis and appropriate treatment can help prevent the condition from worsening and maintain good vision.

**Copilot**
Keratoconus does not go away on its own. It is a chronic condition that typically requires management and treatment to prevent progression and improve vision. While some individuals may experience a stabilization of the condition, it generally does not reverse without medical intervention.<br><br>Treatments like corneal cross-linking can help halt progression, and specialized contact lenses or other surgical options can improve vision. Regular check-ups with your eye doctor are essential to monitor and manage the condition effectively.<br><br>Have you discussed treatment options with your eye specialist?
**Chatsonic**
Keratoconus does not typically resolve or go away on its own. It is a progressive condition characterized by the thinning and bulging of the cornea, leading to visual impairment. While the rate of progression can vary among individuals, keratoconus generally requires ongoing monitoring and management to address changes in corneal shape and visual acuity.<br><br>**Stability and Progression**<br><br>• In some cases, keratoconus progression may stabilize on its own without intervention. However, the condition does not spontaneously resolve, and the irregular corneal shape characteristic of keratoconus typically requires proactive management to maintain vision and corneal health.<br><br>**Management Approaches**<br><br>• Various treatments, such as specialized contact lenses, corneal cross-linking, and surgical interventions, are employed to address keratoconus and prevent further progression.<br><br>**Importance of Monitoring**<br><br>• Regular monitoring by an eye care professional is essential to assess the status of the condition and determine the most appropriate management strategies.<br><br>**Conclusion**<br><br>In summary, keratoconus does not generally resolve without intervention. While some individuals may experience periods of stability, proactive management and ongoing monitoring are crucial to address the progression of the condition and maintain visual function. Early diagnosis and

appropriate treatment can help mitigate the impact of keratoconus on vision and overall eye health.

Perplexity

Keratoconus does not typically go away on its own. It is a progressive condition characterized by the thinning and bulging of the cornea, leading to visual impairment. Here are some key points regarding the nature of keratoconus:

Progressive Nature

- **Progression**: Keratoconus usually begins in adolescence or early adulthood and can progress over time, particularly during the late teenage years and early twenties. The rate of progression varies among individuals, and while some may experience rapid changes, others may have a more stable course.
- **Stabilization**: For many patients, the progression of keratoconus may stabilize after reaching their mid-30s. However, this is not guaranteed, and ongoing monitoring is essential.

Treatment Options

- **Collagen Cross-Linking**: This procedure is designed to halt the progression of keratoconus. It involves applying riboflavin (vitamin B2) to the cornea and exposing it to ultraviolet light, which strengthens the corneal structure. If performed early, it may prevent further deterioration.
- **Contact Lenses**: In the initial stages, glasses may provide adequate vision correction, but as the condition progresses, specialized contact lenses are often required.
- **Surgical Interventions**: In advanced cases, treatments such as corneal transplants may be necessary to restore vision.

Conclusion

While keratoconus is a chronic condition that typically does not resolve on its own, appropriate treatment and management can help stabilize the condition and improve visual outcomes. Regular eye examinations and early intervention are crucial for effectively managing keratoconus and preventing its progression.

Question 18: Can Keratoconus Cause Dry Eye?

ChatGPT-3.5

Yes, keratoconus can be associated with dry eye. Several factors related to keratoconus may contribute to dry eye symptoms:

1. **Irregular Corneal Shape**: The irregular corneal surface in keratoconus can disrupt the even distribution of the tear film across the eye, leading to dryness or discomfort.
2. **Contact Lenses**: Many people with keratoconus rely on rigid gas permeable (RGP), scleral, or hybrid contact lenses for vision correction. These lenses can cause or worsen dry eye by reducing the amount of oxygen that reaches the cornea or disrupting the tear film. Improperly fitted lenses can also contribute to dryness and irritation.
3. **Eye Rubbing**: People with keratoconus often rub their eyes due to discomfort or itchiness, which can worsen dry eye symptoms by irritating the ocular surface and disrupting tear production.
4. **Tear Film Instability**: Keratoconus may cause an imbalance in the tear film due to changes in corneal shape, making it harder for tears to properly lubricate the eye.

Treatments for dry eye in keratoconus patients include using lubricating eye drops, ensuring proper contact lens fit, and addressing any underlying ocular surface issues.

ChatGPT-4.0

Keratoconus can indeed lead to dry eye symptoms due to the irregular shape of the cornea, which disrupts the normal distribution of tears. This disruption can cause increased tear evaporation and instability, resulting in dry eye discomfort.

Detailed Explanation:

1. **Irregular Corneal Surface**: The cone-shaped cornea in keratoconus affects how tears spread across the eye's surface. This irregularity can lead to uneven tear distribution, causing some areas to become dry.
2. **Increased Tear Evaporation**: The abnormal shape can also lead to increased evaporation of tears, as the tear film may not be stable enough to protect the eye adequately.
3. **Symptoms Overlap**: Patients with keratoconus often report symptoms that overlap with dry eye, such as blurred vision, irritation, and sensitivity to light, making it essential to address both conditions.
4. **Management**: It is important for individuals with keratoconus to monitor and manage dry eye symptoms, as untreated dry eye can worsen overall eye health and comfort.

In summary, keratoconus can contribute to dry eye symptoms, and addressing both conditions is crucial for maintaining optimal eye health.
**Gemini**
**Yes, keratoconus can cause dry eye.** The irregular shape of the cornea in keratoconus can disrupt the tear film, leading to dryness, irritation, and discomfort.<br>People with keratoconus may experience dry eye symptoms such as:<br>• **Burning or stinging sensation**<br>• **Feeling like there is something in the eye**<br>• **Redness or dryness**<br>• **Blurred vision**<br>• **Light sensitivity**<br>If you have keratoconus and are experiencing dry eye symptoms, it's important to **see an eye doctor** for proper diagnosis and treatment. Your eye doctor may recommend artificial tears, eye lubricants, or other treatments to help manage your dry eye.<br>**Treating both keratoconus and dry eye is important** to prevent further discomfort and vision problems.
**Copilot**
Yes, keratoconus can indeed cause dry eye symptoms. The irregular shape of the cornea in keratoconus can disrupt the normal distribution of the tear film, leading to reduced tear stability and increased evaporation. This can result in symptoms such as eye discomfort, burning, itching, and redness.<br>Additionally, treatments for keratoconus, like rigid gas-permeable contact lenses, can sometimes exacerbate dry eye symptoms due to reduced oxygen flow to the cornea.<br>If you're experiencing these symptoms, it's important to consult with an eye care professional for proper diagnosis and management. They can recommend treatments to help alleviate dry eye symptoms and manage keratoconus effectively.<br>Is there anything else you'd like to know about keratoconus or dry eye?

**Chatsonic**
Yes, keratoconus can contribute to dry eye symptoms in some individuals. The irregular shape of the cornea associated with keratoconus can disrupt the normal distribution of tears across the ocular surface, leading to dry eye discomfort. Additionally, factors such as increased eye rubbing due to itching or irritation associated with keratoconus can exacerbate dry eye symptoms.<br><br>**Corneal Irregularity**<br><br>• The distortion of the corneal surface in keratoconus can disrupt the even distribution of tears, leading to dry spots on the ocular surface.<br><br>**Reduced Blinking Efficiency**<br><br>• Individuals with keratoconus may blink less efficiently due to discomfort or compromised visual acuity, which can contribute to dry eye symptoms.<br><br>**Increased Eye Rubbing**<br><br>• The urge to rub the eyes, which is common in keratoconus, can disrupt the tear film and exacerbate dry eye symptoms.<br><br>**Contact Lens Use**<br><br>• Contact lens wear, often used to correct vision in keratoconus, can contribute to dry eye symptoms if the lenses are not properly fitted or if there is inadequate tear exchange under the lenses.<br><br>**Conclusion**<br><br>Given these factors, individuals with keratoconus may be more prone to experiencing dry eye symptoms. It's important for individuals with keratoconus to discuss any dry eye discomfort with their eye care professional, as proper management of dry eye symptoms can contribute to overall ocular comfort and well-being.
**Perplexity**
Yes, keratoconus can cause dry eye symptoms. The irregular shape of the cornea associated with keratoconus disrupts the normal tear film, which is essential for keeping the eyes lubricated and comfortable. Here are some key points regarding the relationship between keratoconus and dry eye:

Causes of Dry Eye in Keratoconus

- **Irregular Corneal Surface**: The bulging and thinning of the cornea in keratoconus can lead to an uneven distribution of tears, making it difficult for the eyes to maintain proper lubrication.
- **Contact Lens Use**: Many individuals with keratoconus rely on contact lenses for vision correction. However, wearing contact lenses can further interfere with the eye's ability to lubricate itself, potentially leading to or exacerbating dry eye symptoms.
- **Tear Film Instability**: Patients with keratoconus often have lower tear volumes and reduced tear stability, which can contribute to the sensation of dryness and discomfort.

Symptoms of Dry Eye in Keratoconus

Individuals with keratoconus may experience a range of dry eye symptoms, including:

- Dryness and irritation
- Scratchy or gritty sensations in the eyes
- Blurred vision
- Increased sensitivity to light
- Fatigue or tired eyes

Management Strategies

To manage dry eye symptoms in individuals with keratoconus, several strategies can be employed:

- **Artificial Tears**: Over-the-counter preservative-free artificial tears can help lubricate the eyes and alleviate dryness.
- **Scleral Lenses**: These larger contact lenses vault over the cornea and rest on the sclera, creating a reservoir of fluid that can help relieve dry eye symptoms while providing better vision.
- **Prescription Eye Drops**: In more severe cases, prescription medications such as Restasis or Xiidra may be recommended to increase tear production and reduce inflammation.
- **Punctal Plugs**: These small devices can be inserted into the tear ducts to help retain tears on the ocular surface.
- **Regular Eye Care**: Regular visits to an eye care professional are essential for monitoring both keratoconus and dry eye symptoms, ensuring appropriate treatment adjustments as needed.

In conclusion, keratoconus can indeed cause dry eye symptoms, and effective management is important for improving comfort and maintaining vision quality. If you are experiencing dry eye symptoms alongside keratoconus, it is advisable to consult with an eye care specialist for tailored treatment options.

Question 19: What Do I Do If I Think I Have Keratoconus?

ChatGPT-3.5

If you suspect you have keratoconus, it's important to take action quickly to manage the condition effectively and preserve your vision. Here's what you should do:

1. Schedule an Eye Exam with an Eye Specialist

- Make an appointment with an **ophthalmologist** or **optometrist** who specializes in corneal diseases. They will perform a thorough eye examination and specialized tests, like **corneal topography** and **pachymetry**, to diagnose keratoconus.

2. Monitor Your Symptoms

- Pay attention to changes in your vision, such as increasing blurriness, distorted vision, sensitivity to light, or frequent changes in your prescription. These are signs of keratoconus that you should report to your doctor.

3. Avoid Eye Rubbing

- Since eye rubbing can worsen keratoconus, it's crucial to stop this habit. If your eyes feel itchy or irritated, use lubricating eye drops or consult your doctor for allergy treatment.

4. Explore Treatment Options

- If keratoconus is diagnosed, your eye specialist may recommend treatment options such as:
  - **Glasses or contact lenses** for early stages.
  - **Rigid gas permeable (RGP), hybrid, or scleral lenses** for better vision correction.
  - **Corneal Cross-Linking (CXL)** to stop disease progression in more advanced cases.

5. Follow Up Regularly

- Regular eye checkups are essential to monitor the progression of keratoconus and adjust your treatment as needed.

6. Educate Yourself

- Learn about the condition and treatment options so you can make informed decisions about your eye care.

Early intervention, especially with treatments like **corneal cross-linking (CXL)**, can help prevent the condition from worsening and preserve your vision long-term.

**ChatGPT-4.0**
If you suspect you have keratoconus, it is important to take the following steps:<br>1. **Schedule an Appointment**: Contact your eye care provider to discuss your symptoms and concerns. They will review your medical and family history and conduct a comprehensive eye exam, which may include corneal imaging studies to assess the shape of your cornea 2.<br>2. **Avoid Eye Rubbing**: Try to avoid rubbing your eyes, as this can worsen the condition and lead to further corneal damage 4.<br>3. **Monitor Symptoms**: Be aware of symptoms such as blurred or distorted vision, increased sensitivity to light, and frequent changes in your eyeglass prescription. If you experience a sudden worsening of vision, seek immediate medical attention 3.<br>4. **Discuss Treatment Options**: Your eye doctor may recommend treatments to slow the progression of keratoconus, such as corneal cross-linking, or corrective lenses. In more severe cases, surgery may be necessary 23.<br>5. **Regular Check-ups**: Keep regular appointments with your eye care provider to monitor the condition and adjust treatment as needed 1.
**Gemini**
**If you suspect you have keratoconus, it's important to see an eye doctor as soon as possible.** Early diagnosis and treatment can help prevent vision loss and improve your quality of life.<br>Here are some steps you can take:<br>1. **Make an appointment with an eye doctor:** Schedule a comprehensive eye exam to assess your vision and check for signs of keratoconus.<br>2. **Be honest about your symptoms:** Describe any changes in your vision, such as blurry vision, double vision, or light sensitivity.<br>3. **Provide your family history:** If you have a family history of keratoconus or other eye conditions, be sure to inform your eye doctor.<br>4. **Follow your doctor's recommendations:** If your eye doctor diagnoses keratoconus, follow their treatment plan carefully and attend regular follow-up appointments.

Remember, early diagnosis and treatment can make a significant difference in managing keratoconus and preventing vision loss.

Copilot

If you suspect you have keratoconus, it's important to take the following steps:

1. **Schedule an Eye Exam**: Make an appointment with an eye care professional, such as an optometrist or ophthalmologist. They can perform specialized tests to diagnose keratoconus.
2. **Discuss Symptoms**: Be sure to mention all your symptoms, including any changes in vision, eye discomfort, or dry eye symptoms.
3. **Follow Recommendations**: If diagnosed with keratoconus, your eye care professional will discuss treatment options. These may include:
   - **Specialized Contact Lenses**: Rigid gas-permeable lenses or scleral lenses can help improve vision.
   - **Corneal Cross-Linking**: A procedure to strengthen the cornea and slow the progression of keratoconus.
   - **Intacs**: Small implants inserted into the cornea to help reshape it.
   - **Corneal Transplant**: In severe cases, a corneal transplant may be necessary.
4. **Regular Monitoring**: Regular follow-up appointments are crucial to monitor the condition and adjust treatments as needed.
5. **Manage Dry Eye**: If you experience dry eye symptoms, your eye care professional can recommend treatments such as artificial tears, prescription eye drops, or lifestyle changes to help manage these symptoms.

Taking these steps can help you manage keratoconus effectively and maintain your vision. If you have any more questions or need further information, feel free to ask!

Chatsonic

If you suspect that you may have keratoconus, it's crucial to seek evaluation and guidance from an eye care professional. Here are the steps to consider if you think you have keratoconus:

Schedule an Eye Examination

- Contact an optometrist or ophthalmologist to schedule a comprehensive eye examination. Be sure to communicate your concerns and any visual symptoms you may be experiencing.

Diagnostic Tests

- The eye care professional may perform various tests to assess the shape and condition of your cornea, such as corneal topography and corneal pachymetry. These tests can aid in the diagnosis of keratoconus.

Discussion and Evaluation

- Engage in a thorough discussion with the eye care professional regarding your symptoms, family history, and any relevant medical information. This information can help in the accurate diagnosis of keratoconus.

Treatment Options

- If a diagnosis of keratoconus is confirmed, the eye care professional can discuss the available treatment options, which may include specialized contact lenses, corneal cross-linking, and other interventions to manage the condition and preserve vision.

Ongoing Monitoring

- If diagnosed with keratoconus, regular monitoring by the eye care professional is essential to track the progression of the condition and adjust treatment as needed.

Conclusion

Seeking prompt evaluation and guidance from an eye care professional is essential if you suspect you have keratoconus. Early diagnosis and appropriate management can help address the condition and preserve visual function, emphasizing the importance of proactive eye care in addressing potential ocular conditions such as keratoconus.

Perplexity

If you suspect that you have keratoconus, it’s important to take the following steps to ensure proper diagnosis and management:

1. Schedule an Appointment with an Eye Care Professional

- **Consult an Eye Doctor**: Make an appointment with an ophthalmologist or optometrist who specializes in corneal diseases. They will conduct a thorough examination to assess your eye health and determine if keratoconus is present.

2. Undergo Comprehensive Eye Exams

- **Visual Acuity Tests**: These tests will evaluate how well you see at various distances.
- **Corneal Topography**: This is a key diagnostic tool that creates a detailed map of the curvature of your cornea, helping to identify irregularities characteristic of keratoconus.
- **Slit-Lamp Examination**: This allows the doctor to examine the front structures of your eye, including the cornea, for any signs of keratoconus.
- **Pachymetry**: This test measures the thickness of the cornea, which can help in diagnosing keratoconus.

3. Discuss Symptoms

- **Report Symptoms**: Be prepared to discuss any symptoms you are experiencing, such as blurred or distorted vision, frequent changes in your glasses prescription, light sensitivity, and difficulty seeing at night.

4. Explore Treatment Options

- **Understand Treatment Plans**: If diagnosed with keratoconus, your eye care professional will discuss potential treatment options based on the severity of your condition. These may include:
  - **Eyeglasses or Soft Contact Lenses**: Often sufficient in the early stages.
  - **Rigid Gas Permeable Contact Lenses**: These provide better vision correction as the disease progresses.
  - **Scleral Lenses**: These larger lenses can improve comfort and vision by vaulting over the cornea.
  - **Corneal Collagen Cross-Linking (CXL)**: A procedure to strengthen the cornea and slow progression.
  - **Surgical Options**: In advanced cases, corneal transplant surgery may be considered.

5. Monitor and Follow Up

- **Regular Check-Ups**: After diagnosis, regular follow-up appointments are essential to monitor the condition and adjust treatment as necessary.

Taking these steps can help you manage keratoconus effectively and maintain the best possible vision. Early detection and intervention are crucial for slowing the progression of the disease and preserving eye health.

Question 20: What Should Be Considered After Keratoconus Surgery?

ChatGPT-3.5
After keratoconus surgery, proper care and follow-up are crucial for a smooth recovery and to ensure the best possible outcome. Here are important things to consider:<br><br>**1. Follow Post-Operative Care Instructions**<br><br>• Your surgeon will provide specific guidelines for caring for your eyes after surgery. These may include using prescribed medications, such as antibiotic or anti-inflammatory eye drops, to prevent infection and reduce inflammation.<br><br>**2. Avoid Eye Rubbing**<br><br>• Eye rubbing can disrupt the healing process and potentially damage the cornea, especially after procedures like **corneal cross-linking (CXL)** or **corneal transplant**. Be mindful of this during the recovery period.<br><br>**3. Monitor for Complications**<br><br>• Watch for any unusual symptoms such as pain, excessive redness, sudden vision changes, or discharge, as these could indicate complications like infection, inflammation, or graft rejection (in the case of a corneal transplant). Contact your doctor immediately if you notice any of these signs.<br><br>**4. Follow Up with Your Doctor**<br><br>• Regular follow-up visits are essential to monitor healing and ensure the surgery's success. Your doctor will assess your cornea's recovery and make any necessary adjustments to your treatment plan.<br><br>**5. Vision Changes**<br><br>• It may take time for your vision to stabilize after surgery. Some procedures, like corneal transplants, can require several months to a year for vision to fully improve. You may need to be fitted with glasses or contact lenses during this time.<br><br>**6. Limit Strenuous Activity**<br><br>• Avoid heavy lifting, vigorous exercise, or any activities that could strain your eyes in the initial weeks after surgery, as this can impact healing.<br><br>**7. Protect Your Eyes**<br><br>• Wear sunglasses to protect your eyes from UV light and avoid exposure to dust, wind, or other irritants that can slow down recovery. Your doctor may also recommend protective eyewear, especially if you've had a corneal transplant.<br><br>**8. Be Patient**

- Full recovery after certain keratoconus surgeries, like **corneal transplants**, can take time. Be patient with the healing process and follow your doctor's advice for the best long-term results.

Adhering to these considerations helps ensure a successful outcome and reduces the risk of complications following keratoconus surgery.

ChatGPT-4.0

The information on post-surgery considerations for keratoconus has been clearly communicated, covering essential aspects such as pain management, epithelial healing, infection prevention, regular monitoring, visual rehabilitation, and avoiding irritants. Each point has been explained in detail to ensure understanding. I will now summarize these considerations in a concise format for easy reference.

Post-Surgery Considerations for Keratoconus

1. **Pain Management**:

- Mild to moderate discomfort is common post-surgery.
- Over-the-counter pain relievers are typically recommended, with stronger medications available if needed.

2. **Epithelial Healing**:

- Proper healing of the corneal epithelium is critical.
- A bandage contact lens is often used to protect the cornea during recovery.

3. **Infection Prevention**:

- Topical antibiotics (e.g., fluoroquinolones) are prescribed to prevent infections.
- Patients should avoid exposing their eyes to water and maintain hygiene.

4. **Regular Monitoring**:

- Follow-up visits with an ophthalmologist are essential to assess healing and detect complications early.

5. **Visual Rehabilitation**:

- Patients may require corrective lenses post-surgery to enhance vision.
- Regular assessments help determine the best options for visual correction.

6. **Avoiding Irritants**:

- Patients should avoid environments with dust, smoke, or strong winds.
- Protective eyewear, such as sunglasses, is recommended to shield the eyes.

Gemini

After keratoconus surgery, it's important to follow your eye doctor's post-operative instructions carefully to ensure a successful recovery. Here are some key considerations:

- **Rest:** Avoid strenuous activities or heavy lifting for the recommended period.
- **Eye care:** Use prescribed eye drops or ointments as directed.
- **Avoid irritants:** Protect your eyes from dust, wind, and other irritants.
- **Follow-up appointments:** Attend all scheduled follow-up appointments with your eye doctor to monitor your healing progress.
- **Avoid water:** Keep your eyes dry and avoid swimming or showering for the recommended period.
- **Wear protective eyewear:** Wear sunglasses or protective eyewear when outdoors.
- **Limit screen time:** Reduce screen time, especially activities that require prolonged focus, such as reading or using a computer.

Remember, these are general guidelines. Your eye doctor will provide specific instructions based on your individual situation and the type of surgery you underwent. It's essential to follow their recommendations closely to optimize your recovery and minimize the risk of complications.

Copilot

After keratoconus surgery, there are several important considerations to ensure proper healing and optimal outcomes:

1. **Follow Post-Operative Instructions**: Adhere strictly to the guidelines provided by your surgeon. This includes using prescribed medications, such as antibiotic and anti-inflammatory eye drops, to prevent infection and reduce inflammation.
2. **Attend Follow-Up Appointments**: Regular check-ups are crucial to monitor the healing process and detect any complications early. Your eye care professional will assess your progress and make any necessary adjustments to your treatment plan.

3. **Protect Your Eyes**: Avoid rubbing your eyes, as this can interfere with healing. Wear protective eyewear, especially in environments where your eyes might be exposed to dust or debris.
4. **Manage Dry Eye Symptoms**: Post-surgery, you might experience dry eye symptoms. Use artificial tears or other recommended treatments to keep your eyes lubricated and comfortable.
5. **Avoid Strenuous Activities**: Refrain from heavy lifting, vigorous exercise, or activities that could strain your eyes for the period recommended by your surgeon.
6. **Monitor for Symptoms**: Be vigilant for any signs of complications, such as increased pain, redness, vision changes, or discharge. Contact your eye care professional immediately if you notice any of these symptoms.
7. **Gradual Return to Normal Activities**: Follow your surgeon's advice on when to resume normal activities, including work, driving, and using digital devices.
8. **Healthy Lifestyle**: Maintain a healthy diet and stay hydrated to support overall eye health and recovery.

By carefully following these steps, you can help ensure a smooth recovery and the best possible outcome from your keratoconus surgery. If you have any specific concerns or questions, don't hesitate to reach out to your eye care professional.

Chatsonic

After undergoing keratoconus surgery, it's important to consider several factors to ensure proper recovery and ongoing eye health. Here are some key considerations:

Postoperative Care

- Adhere to the postoperative care instructions provided by your surgeon, which may include the use of prescription eye drops, avoiding rubbing the eyes, and attending follow-up appointments.

Visual Recovery

- Understand that visual recovery after keratoconus surgery may take time, and your vision may initially be blurry or fluctuate as the eyes heal. Be patient and follow up with your surgeon regarding any concerns about your vision.

Protection and Avoidance of Irritants

- Protect your eyes from irritants, such as dust, wind, and direct sunlight. Use sunglasses and avoid activities that could expose your eyes to potential injury during the healing period.

Monitoring for Complications

- Be vigilant for any signs of complications, such as increased pain, redness, or changes in vision, and promptly report any concerning symptoms to your surgeon.

Long-Term Follow-Up

- Maintain regular follow-up appointments with your surgeon to monitor the long-term outcomes of the surgery and address any potential changes in your vision or ocular health.

Conclusion

After keratoconus surgery, attentive postoperative care and ongoing communication with your surgeon are essential for ensuring proper healing, monitoring visual recovery, and addressing any potential concerns. By following the recommended guidelines and maintaining regular follow-up, you can optimize the outcomes of the surgery and promote the long-term health of your eyes.

Perplexity

After undergoing keratoconus surgery, such as corneal collagen cross-linking (CXL) or a corneal transplant, several important considerations should be taken into account to ensure a smooth recovery and optimal outcomes:

Postoperative Care

1. **Eye Protection**:

- **Bandage Contact Lens**: A bandage contact lens may be placed on the eye to protect the cornea and aid in healing. It helps reduce discomfort and supports the regeneration of the corneal surface.

2. **Medication**:

- **Antibiotics and Anti-inflammatory Drops**: Patients are typically prescribed antibiotic and anti-inflammatory eye drops to prevent infection and control inflammation. It's crucial to follow the prescribed regimen closely.

3. **Pain Management**:

- **Oral Analgesics**: Pain relief medication may be recommended, especially in the first few days post-surgery. Managing discomfort is important for a smoother recovery.

4. **Avoid Rubbing**:

- Patients should avoid rubbing their eyes to prevent irritation or damage to the cornea during the healing process.

5. **Activity Restrictions**:

- **Avoid Water Exposure**: It's important to avoid swimming or exposing the eyes to water for a specific period to reduce the risk of infection. Follow your surgeon's advice regarding activity restrictions.

Follow-Up Visits

- **Regular Monitoring**: Schedule follow-up appointments with your ophthalmologist to monitor the healing process and detect any potential complications early. These visits are crucial for assessing the success of the surgery and making any necessary adjustments to the treatment plan.

Visual Rehabilitation

1. **Eyeglasses or Contact Lenses**:

- After surgery, patients may require new eyeglasses or specialized contact lenses to correct refractive errors as the cornea heals. Vision improvement may take time, so patience is essential.

2. **Gradual Vision Improvement**:

- Understand that vision may fluctuate and may not improve immediately after surgery. It can take several months for vision to stabilize as the eye heals.

Lifestyle Considerations

- **Avoid Eye Strain**: Take breaks from screens and practice the 20-20-20 rule to reduce eye strain: every 20 minutes, look at something 20 feet away for 20 seconds.
- **Healthy Diet**: Maintain a balanced diet rich in fruits, vegetables, and whole grains to support overall eye health.

Conclusion

Postoperative care after keratoconus surgery is critical for successful recovery and long-term visual outcomes. Following your surgeon's instructions, attending follow-up appointments, and making necessary lifestyle adjustments will help ensure the best possible results. If you have any concerns during your recovery, do not hesitate to contact your eye care professional for guidance.
